# A Photo-Crosslinking Approach to Identify Class II SUMO-1 Binders

**DOI:** 10.3389/fchem.2022.900989

**Published:** 2022-05-30

**Authors:** Kira Brüninghoff, Stephanie Wulff, Wolfgang Dörner, Ruth Geiss-Friedlander, Henning D. Mootz

**Affiliations:** ^1^ Institute of Biochemistry, University of Münster, Münster, Germany; ^2^ Institute of Molecular Medicine and Cell Research, University of Freiburg, Freiburg, Germany

**Keywords:** genetic code expansion, chemical crosslinking, mass spectrometry, p-benzoyl phenylalanine, ubiquitin-like protein

## Abstract

The small ubiquitin-like modifier (SUMO) is involved in various cellular processes and mediates known non-covalent protein-protein interactions by three distinct binding surfaces, whose interactions are termed class I to class III. While interactors for the class I interaction, which involves binding of a SUMO-interacting motif (SIM) to a hydrophobic groove in SUMO-1 and SUMO-2/3, are widely abundant, only a couple of examples have been reported for the other two types of interactions. Class II binding is conveyed by the E67 loop region on SUMO-1. Many previous studies to identify SUMO binders using pull-down or microarray approaches did not strategize on the SUMO binding mode. Identification of SUMO binding partners is further complicated due to the typically transient and low affinity interactions with the modifier. Here we aimed to identify SUMO-1 binders selectively enriched for class II binding. Using a genetically encoded photo-crosslinker approach, we have designed SUMO-1 probes to covalently capture class II SUMO-1 interactors by strategically positioning the photo-crosslinking moiety on the SUMO-1 surface. The probes were validated using known class II and class I binding partners. We utilized the probe with p-benzoyl-phenylalanine (BzF, also termed BpF or Bpa) at the position of Gln69 to identify binding proteins from mammalian cell extracts using mass spectrometry. By comparison with results obtained with a similarly designed SUMO-1 probe to target SIM-mediated binders of the class I type, we identified 192 and 96 proteins specifically enriched by either probe, respectively. The implicated preferential class I or class II binding modes of these proteins will further contribute to unveiling the complex interplay of SUMO-1-mediated interactions.

## Introduction

Posttranslational modification of thousands of proteins by the small ubiquitin-like modifier SUMO affects many cellular processes such as cell cycle regulation, replication or DNA repair (Flotho and Melchior, 2013; Hay, 2013; Hendriks and Vertegaal, 2016; Chang and Yeh, 2020). In mammalian cells, SUMO-1, SUMO-2 and SUMO-3 are the major SUMO paralogs, with SUMO-1 sharing approximately 45% sequence identity with the highly similar SUMO-2/3 (Saitoh and Hinchey, 2000). SUMOylation of proteins requires an enzymatic cascade consisting of a dimeric E1 activating enzyme (SAE1/SAE2, also termed AOS1/UBA2), an E2 conjugating enzyme (Ubc9) and an E3 ligase (a few are known, e.g., RanBP2 and ZNF451) leading to isopeptide bond formation between the C-terminal di-glycine motif of SUMO and a lysine residue in the substrate protein. SUMOylation is highly dynamic due to the activity of SUMO specific proteases (SENP) that deconjugate SUMO from target proteins (Pichler et al., 2017).

**FIGURE 1:**
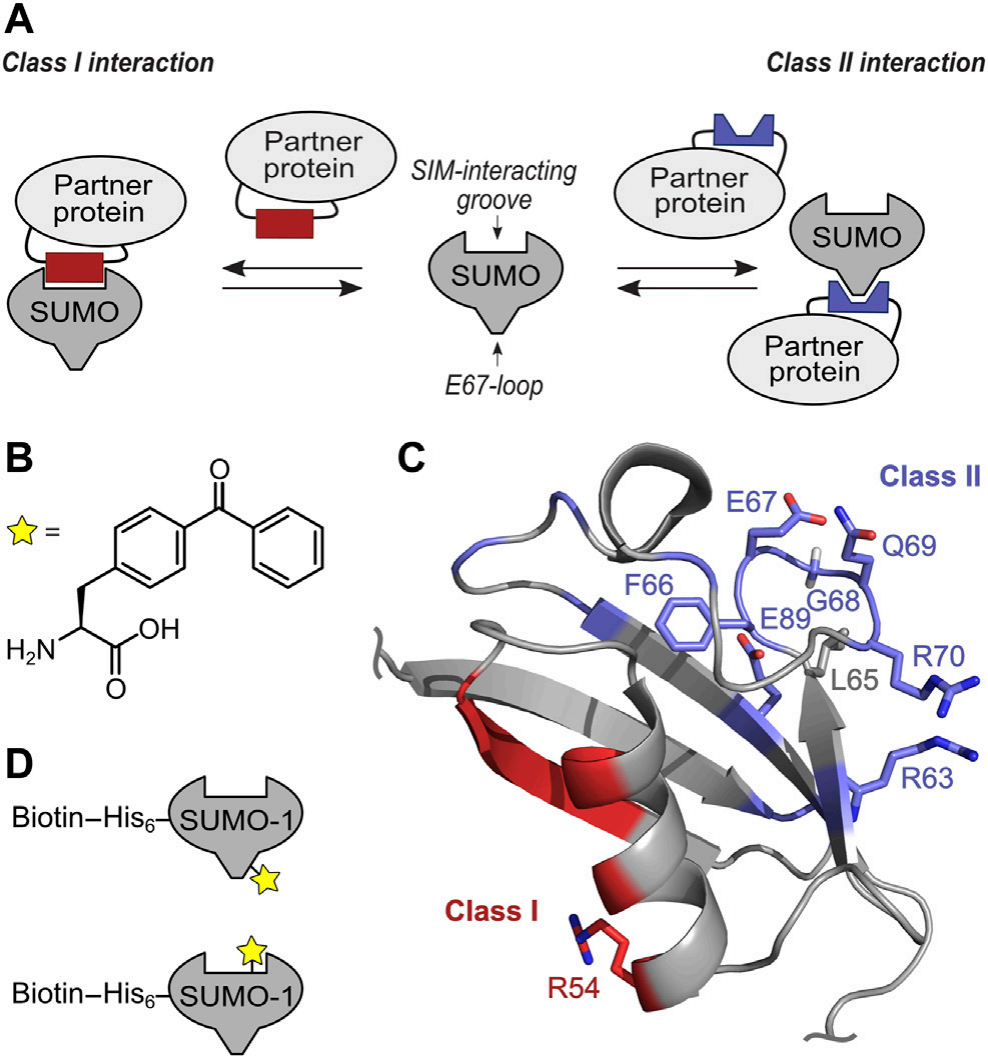
Concept of targeting class I and II SUMO Interaction interface with selective SUMO-1 photo-crosslinking probes. **(A)** Scheme of SUMO-protein interaction using SIM interacting groove and E67-loop binding. **(B)** Structure of unnatural amino acid BzF utilized as photo-crosslinker. **(C)** NMR structure (pdb-code: 1A5R) of SUMO-1. Residues involved in class II interaction are highlighted in blue and the positions tested for BzF incorporation are shown as sticks. Residues forming the class I interaction site are shown in red and the R54 side chain used to incorporate BzF is highlighted in sticks representation. **(D)** Scheme of the mono-SUMO-1 probes containing BzF close to the E67 loop (top) and at R54 close to the SIM binding groove (bottom). Biotin was conjugated to a single cysteine in an N-terminal extension *via* maleimide chemistry.

At a molecular level, SUMOylation of target proteins typically affects protein-protein interactions by either covering existing binding sites or by creating new specific interaction sites (Geiss-Friedlander and Melchior, 2007). Three different interaction surfaces have been characterized on SUMO, class I to class III, these participate in non-covalent protein-protein interactions in a partly paralog specific way (Pichler et al., 2017). The best-characterized interaction surface of SUMO represents a hydrophobic groove formed by the second SUMO β-sheet and the α-helix (class I), which binds a SUMO-interacting motif (SIM) of interaction partners. SIMs are short peptide sequences consisting of three to four hydrophobic amino acids flanked C-or N-terminally by one or more acidic residues (Song et al., 2004; Hecker et al., 2006; Kerscher, 2007; Praefcke et al., 2012; Kötter et al., 2019). The affinity of SIM to SUMO is moderate to weak with dissociation constants between 1–100 µM. SUMO-SIM interactions are often involved in the formation of bigger protein complexes in which several SIMs can bind multi- or polySUMOylated substrates and additive or cooperative effects may play a role (Baba et al., 2005; Psakhye and Jentsch, 2012; Keusekotten et al., 2014; Aguilar-Martinez et al., 2015). The class II SUMO interaction employs a binding surface opposite of the SIM binding groove, namely a loop region connecting the third and fourth SUMO β-sheet termed the E67 interacting loop (EIL) (Figure 1A) (Pilla et al., 2012; Brantis-de-Carvalho et al., 2015; Pichler et al., 2017; Sriramachandran et al., 2019). To date, only a few interaction partners for the class II surface are known. The backside of Ubc9 and the dipeptidyl peptidase 9 (DPP9) bind here in different ways. In DPP9, an arm-like elongation was identified that specifically binds SUMO-1 but not SUMO-2/3 (Pilla et al., 2012), whereas the Ubc9-SUMO interaction is independent of paralogs (Liu et al., 1999; Capili and Lima, 2007; Duda et al., 2007; Knipscheer et al., 2007). Recently, Arkadia/RNF111 was found to be able to bind the class II interaction site by its SUMO-1 specific binding site which shares sequence similarities to the SUMO-1 binding motif in DPP9 (Sriramachandran et al., 2019). Finally, proteins identified as class III interaction partners bind *via* a ZZ zinc finger domain to a distinct surface of SUMO-1 (Danielsen et al., 2012; Diehl et al., 2016).

It remains unknown if more proteins interact with SUMO through the class II and class III interfaces or even through so far unknown regions on the surface of the small modifier and what roles these interactions play in cellular pathways. Systematic studies to identify new SUMO interactors using pull-down, yeast-two-hybrid or protein microarrays typically lead to new protein candidates, however, this data does not directly indicate the respective SUMO binding mode (Hannich et al., 2005; Hecker et al., 2006; Aguilar-Martinez et al., 2015; Cox et al., 2017; González-Prieto et al., 2021). Given the transient nature and mostly low binding affinities of non-covalent interaction partners, SUMO binders might be lost during the identification procedure.

Chemical crosslinking fixes non-covalent interactions with a stable bond. Photo-activated crosslinkers generate highly reactive and short-lived intermediates that can insert into bonds in close vicinity (Tanaka et al., 2008; Mishra et al., 2020). Genetically encoded photo-crosslinking amino acids can be incorporated site-specifically into recombinant proteins *via* amber stop codon suppression. A widely used example is para-benzoyl-phenylalanine (BzF, also termed BpF or Bpa) (Figure 1B), which upon irradiation with light of approx. 360–365 nm can be repeatedly activated to a short-lived diradical that inserts into C-H bonds in close spatial proximity on the backbone or side chains (Dormán et al., 2016). A preference for the methionine side chains has been observed (Wittelsberger et al., 2006). Due to flexibility and rotations of the BzF chain and the backbone, distances beyond the initially determined reaction radius of 3.1 Å can be reached (Winnik, 1981; Wittelsberger et al., 2008; Belsom et al., 2017).

We recently reported the usage of genetically encoded photo-crosslinkers to capture and identify SUMO-SIM interactions. Incorporation at position R54 in SUMO-1 was determined as most suitable to capture proteins that bind *via* a SIM to SUMO-1 (Taupitz et al., 2017). R54 is located at the outer edge of the SIM binding groove and is opposite of the class II and class III interaction surfaces of SUMO-1 (Figure 1C). Using the structurally equivalent position R50 in SUMO-2 we also generated mono-, di- and tetra-SUMO-2 (R50BzF) to crosslink and identify 329 SUMO-2 binding partners by LC-MS/MS (Brüninghoff et al., 2020). As expected from the molecular design of the capturing probe, the identified proteins were enriched for containing one or multiple SIM(s). However, as the photo-crosslinking mechanism relies on spatial proximity to the interacting proteins, it was not surprising that with this approach we also enriched proteins that are not thought to directly engage in a SUMO interaction, but may have been close enough to the photo-active moiety to become captured. An example for this case was the identified protein SAE1, that we believe was hit by the crosslinker due to its spatial proximity to the SUMO probe as a result of complex formation with the SIM of UBA2, with which SAE1 forms the heterodimeric E1 SUMO activating enzyme (Brüninghoff et al., 2020). As components of protein complexes interacting with SUMO-modified proteins, such indirect interactors can also be of great interest.

In this study we expanded the photo-crosslinker approach to identify SUMO binders that are candidates to employ the class II SUMO interaction surface of SUMO-1. We first established Q69 as a suitable position for the photo-crosslinking amino acid BzF in SUMO-1 close to the E67-loop that specifically crosslinked with DPP9 and Ubc9. We then employed the SUMO-1 (Q69BzF) probe for identification of binding partners from a mammalian cell extract. Comparison with interactors determined with the SUMO-1 (R54BzF) probe unveiled two sets of proteins with obvious preferences for each of the SUMO binding surfaces. These sets, as well as proteins found to be enriched with both SUMO-1 crosslink probes represent SUMO-1 binders of great confidence either interacting directly with SUMO-1 or being part of SUMO-mediated protein complexes.

## Results and Discussion

### Design Considerations for a Class II SUMO-1 Photo-Crosslinking Probe

We aimed to identify SUMO-1 binding partners from mammalian cell extracts that are likely candidates to employ the class II interaction interface by developing a novel SUMO-1 probe with a strategically positioned photo-crosslinker. Furthermore, we hypothesized that comparison with proteins identified from photo-crosslinking with the previously reported SUMO-1 (R54BzF) probe (Taupitz et al., 2017), directed at binding partners employing the SIM binding groove, should indicate how exclusive the binding partner is for the class II or class I interface (Figure 1D).

To design the SUMO-1 photo-crosslinking probe directed at class II binding partners, we analyzed positions in and around the EIL motif for the class II interaction interface observed in the DPP9-SUMO-1 interaction (Pilla et al., 2012) and in the crystal structure capturing non-covalent backside binding of SUMO-1 by Ubc9 (Capili and Lima, 2007; Knipscheer et al., 2007). Specifically, we chose positions F66, E67, G68, Q69 and R70 for BzF incorporation. Additionally, we tested R63BzF and E89BzF, which are also thought to participate in the binding of DPP9 and Ubc9, respectively (Capili and Lima, 2007; Knipscheer et al., 2007; Pilla et al., 2012). The SUMO-1 (R54BzF) was previously established as a probe to capture SIM-containing proteins that engage the SIM binding groove (Taupitz et al., 2017) and was intended to serve as a class I comparison. We used L65BzF as a negative control. Although L65 is in the vicinity of the EIL loop, this residue is believed to constitute a functionally unrelated surface area of SUMO-1 that is distinct from both the class I and class II regions. To incorporate the unnatural amino acids by the genetic code expansion technology (Liu and Schultz, 2010), an amber stop codon was introduced at the respective positions by site-directed mutagenesis. The different SUMO-1 mutants were expressed in *E. coli* together with an orthogonal pair of aminoacyl-tRNA-synthetase specific for BzF and a cognate tRNA (Chin et al., 2002), and purified by Ni-NTA chromatography *via* their N-terminal His-tag and size exclusion chromatography (Supplementary Figure S1).

### SUMO-1(Q69BzF) Shows the Best Photo-Crosslinking Ratio Between Class II and Class I Binding Partners

**FIGURE 2:**
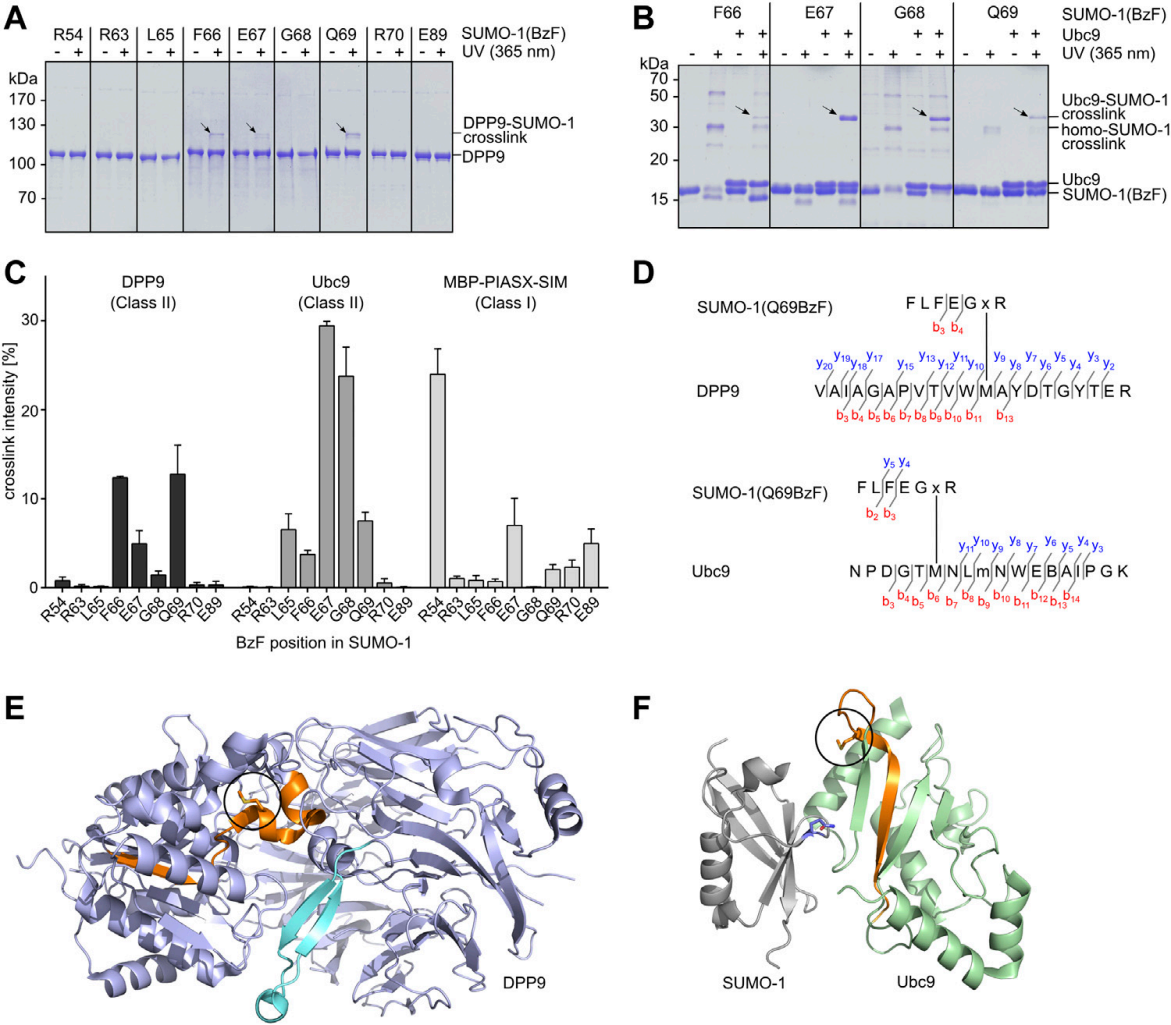
Establishing SUMO-1 photo-crosslinking probes for class II interaction partners. **(A)** and **(B)** SUMO-1 probes with BzF at different positions along the E67-loop were incubated with DPP9 or Ubc9, respectively. Samples before and after UV-irradiation were analyzed by SDS-PAGE and Coomassie-staining. For complete SDS-Gels see Supplementary Figure S6. **(C)** Densitometric analysis of crosslink efficiencies using the binding partners DPP9, Ubc9 and MBP-PIASX-SIM for crosslinking, respectively. Experiments were performed in triplicate. **(D)** Tandem MS mapping of photo-crosslinks obtained for SUMO-1 (Q69BzF) with DPP9 (top) and with Ubc9 (bottom). Depicted in each case is one of the most likely crosslink positions. “x” represents BzF in SUMO-1 peptide. “m” indicates oxidized methionine. See Supplementary Figure S3 for MS/MS spectra. **(E)** Visualization of crosslinked peptide (highlighted in orange) in X-ray structure of DPP9 (pdb-code: 6EOQ). The crosslinked methionine is circled in black. **(F)** Crystal structure of Ubc9 in complex with SUMO-1 (pdb-code: 2UYZ) to visualize the crosslink of SUMO-1 (Q69BzF) with Ubc9. The crosslinked peptide is highlighted in orange and the methionine position is circled in black in the Ubc9 structure. Q69 in SUMO-1 is shown in sticks representation and in blue.

We tested the capability of the SUMO-1(BzF) probes to photo-crosslink the known class II binding partners DPP9 and Ubc9 as purified proteins. We produced DPP9 as a recombinant protein in insect cells and Ubc9 in *E. coli* cells. Protein mixtures were UV-irradiated (λ = 365 nm, 60 min, 8 W) and then analyzed by SDS-PAGE. Figure 2A shows photo-crosslinking assays with DPP9, in which SUMO-1 probes with F66BzF, E67BzF and Q69BzF led to the appearance of a new band on SDS-PAGE consistent with a DPP9-SUMO-1 crosslink product. Note that DPP9 with a calculated molecular weight of 98 kDa migrates in SDS gels at about 110 kDa. The DPP9-SUMO-1 crosslink product (calculated ca. 110 kDa) was observed at 120 kDa. We noted only a very faint crosslink of DPP9 with the R54BzF probe, indicating a specific capture of DPP9 due to SUMO-1 class II binding. Using Ubc9 as binding partner, we observed irradiation-dependent crosslink bands with the L65BzF, F66BzF, E67BzF, G68BzF and Q69BzF probes that were missing in irradiation experiments of Ubc9 alone, suggesting they were specific Ubc9-SUMO-1 crosslink products (Figure 2B; see Supplementary Figure S2A for the remaining positions and the Ubc9 control). We also found significant self-crosslinking of the SUMO-1 (R54BzF), SUMO-1(F66BzF) and SUMO-1 (G68BzF) probes to form putative dimers and multimers, as bands of ca. 30 and 60 kDa were observed in the absence of binding partner. These self-crosslink bands were not visible in the assays with DPP9 due to the different molecular weight range of the SDS-gel.

To further evaluate the selectivity of the novel SUMO-1 probes for class II interaction partners we performed photo-crosslinking assays with a purified class I binding protein for comparison. To this end, we selected the PIASX SIM-sequence, C-terminally fused to maltose-binding protein (MPB) and equipped with a C-terminal His_6_-tag. We detected very faint crosslink bands when combining this protein with the SUMO-1 probes with R63BzF, L65BzF, F66BzF, Q69BzF and R70BzF, and slightly stronger crosslinks seemed to occur with the E67BzF and E89BzF probes (Supplementary Figure S2B and Figure 2C). As expected, the SUMO-1 (R54BzF) probe gave rise to a much more pronounced crosslink band with its cognate class I interaction partner (Supplementary Figure S2B). Figure 2C shows a quantitative comparison of all photo-crosslink efficiencies as determined by densitometric analysis of the protein bands. Together, these data reveal a clear preference of most of the novel SUMO-1 probes for the class II interaction partners, with SUMO-1 (Q69BzF) and SUMO-1(F66BzF) showing the most favorable profile in terms of class specificity as well as efficiency towards both DPP9 and Ubc9.

To further confirm that the covalent crosslinks occurred within the desired class II interaction interface, we mapped the crosslink positions of SUMO-1 (Q69BzF) in DPP9 and Ubc9. We excised the respective crosslink bands from the SDS gels and performed an in-gel tryptic digest, followed by LC-MS/MS analysis. Figure 2D shows the identified crosslinked tryptic peptides of DPP9 and Ubc9, which are structurally located in close proximity to the respective expected SUMO-1 interaction regions in these proteins (Figures 2E,F; see Supplementary Figure S3 for the corresponding MS/MS spectra). These findings are consistent with the idea that close proximity is the crucial parameter for a photo-crosslink to occur. Notably, in both cases one of the likely crosslink positions was a methionine residue, which would be in agreement with a documented higher efficiency of BzF to form crosslinks with this side chain (Wittelsberger et al., 2006). A similar analysis with the F66BzF and E67BzF probes further corroborated our molecular design (Supplementary Figure S4).

**FIGURE 3:**
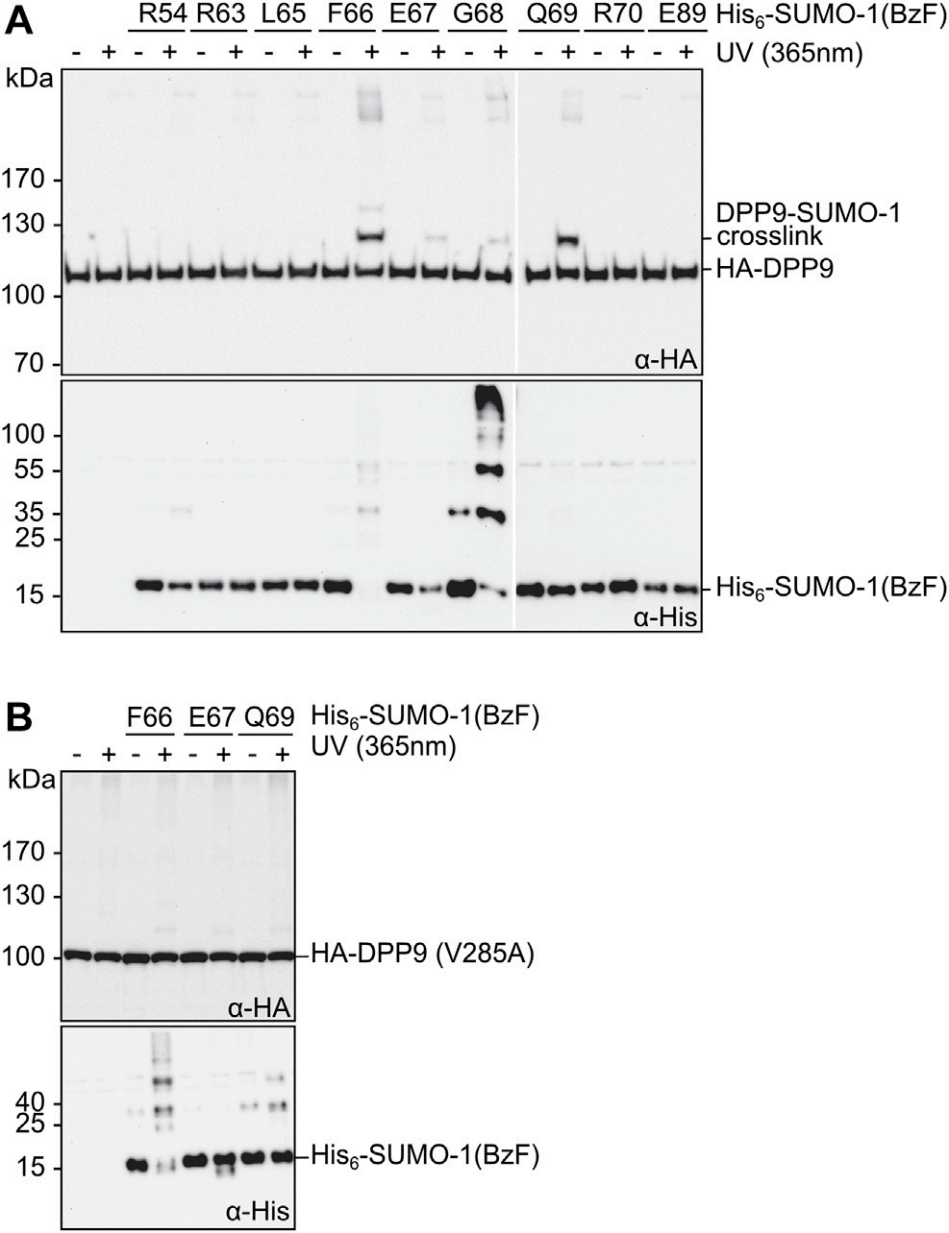
Photo-crosslinking of SUMO-1 probes in mammalian cell extract. **(A)** and **(B)** SUMO-1 probes with BzF at different positions were incubated with HEK293T cell extract of cells transiently transfected with HA-DPP9 and HA-DPP9 (V285A), respectively. Samples taken before and after the UV-irradiation were analyzed by western blotting. The SDS gels used for the immunoblotting in the top panels contained 8% polyacrylamide, whereas those in the bottom panels (intended as loading controls for SUMO-1 probes) were prepared with 17.5% polyacrylamide and hence had limited ability to transfer high molecular weight proteins.

Next, we evaluated the SUMO-1 probes for specific photo-crosslinking of DPP9 in mammalian cells lysates. We transiently transfected HEK293T cells to express HA-DPP9, prepared cell extracts thereof, which were then mixed with the different crosslink probes and exposed to UV-irradiation. In excellent agreement with our above results using purified DPP9, we observed prominent crosslinks with the SUMO-1 probes with F66BzF and Q69BzF (Figure 3A, top panel). An anti-His immunoblot was performed as a loading control for the His-tagged SUMO-1 probes (Figure 3A, bottom panel). Of note, the high-percentage polyacrylamide gel (17.5%) required to visualize the SUMO-1 probe was not suited for efficient transfer of high-molecular weight proteins, which likely explains why the DPP9-SUMO-1 crosslink was not picked up by the anti-His antibody. In a control experiment using a HA-DPP9(V285A) mutant under the same conditions we could only detect much weaker photo-crosslinks with the SUMO-1 probes (Figure 3B, top panel). This result is consistent with previous findings about the impairment of the V285 mutation on the interaction with SUMO-1 in pull-down assays (Pilla et al., 2012), and thus further corroborated the intended design of the SUMO-1 probes as reporters for the SUMO-1 class II interaction.

Based on the above-described results we chose Q69BzF as the most suitable photo-crosslinker position. The SUMO-1 (Q69BzF) probe showed the best ratio between capturing the known class II interaction partners and exhibiting relatively low crosslinking of SIM-mediated binders. Although ultimately one of the other probes might still be valuable to capture new interaction partners, this choice was straight-forward from our experiments to identify new proteins from mammalian cell extracts that bind close to the EIL surface of SUMO-1.

### Identification of Class II SUMO-1 Binding Candidates From Mammalian Cell Extract

Finally, we applied the SUMO-1 (Q69BzF) probe to enrich and identify novel candidates for class II SUMO-1 binding from whole-cell extracts of HeLa cells. In a separate experiment, we also applied the SUMO-1 (R54BzF) probe to possibly differentiate the enriched proteins by their preferred class II binding mode relative to the class I interaction interface. Notably, the covalent bond formation with interacting proteins by photo-crosslinking allows the operation of a highly stringent washing procedure using denaturing conditions (8 M urea) to eliminate unspecifically bound and non-crosslinked proteins (Brüninghoff et al., 2020). Furthermore, very weakly binding proteins, that could otherwise easily and unintentionally be washed away, will also be enriched once they are covalently fixed. To enable pulldowns on streptavidin beads, the above described Q69BzF and R54BzF SUMO-1 probes were bioconjugated with a biotin moiety to a single cysteine introduced into the N-terminal extension of SUMO-1, while the native cysteine at position 52 was replaced by alanine.

**FIGURE 4:**
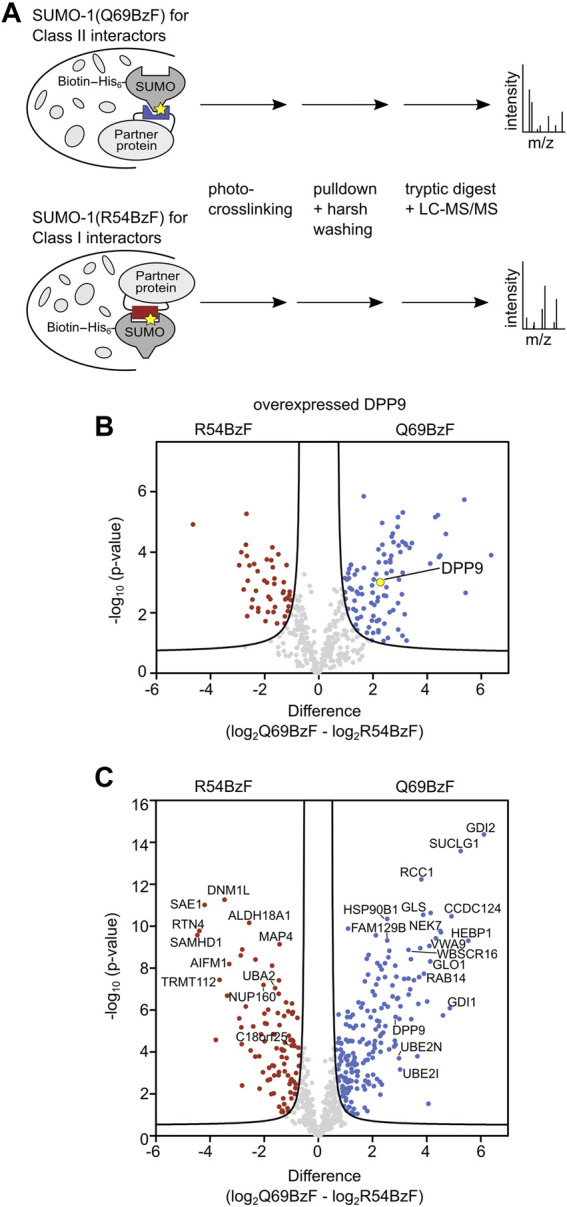
Enrichment and identification of SUMO-1 binding partners. **(A)** Scheme of enrichment protocol. The respective SUMO-1 probe was mixed with whole-cell extracts and the sample was UV-irradiated. The crosslinked products were enriched by streptavidin beads and stringent washing with 8 M Urea was applied, followed by on-bead tryptic digest and LC-MS/MS analysis. Label-free quantification (LFQ) was performed. **(B)** Volcano plot showing DPP9 being significantly enriched by SUMO-1 (Q69BzF) compared to SUMO-1 (R54BzF). The respective SUMO-1 probe was incubated with HEK293T cell extract of cells transiently transfected with HA-DPP9 followed by crosslinking and enrichment as described in **(A)**. Student’s t-test (s0 = 0.5, FDR<0.01, *n* = 3) was applied to determine significantly enriched proteins for each SUMO-1 probe, highlighted in red (R54BzF) and blue (Q69BzF). DPP9 is highlighted as yellow dot. **(C)** Volcano plot visualizing significantly enriched proteins by the two SUMO-1 probes after photo-crosslinking. Enrichment of SUMO-1 binders was performed at endogenous levels from whole-cell HeLa cell extracts. LC-MS/MS and LFQ analysis was performed, followed by Student’s t-test (s0 = 0.5, FDR<0.01, *n* = 6). Significantly enriched proteins are highlighted in red (R54BzF) and blue (Q69BzF).

We first aimed to verify that discrimination of enriched DPP9 according to its preferred class II binding mode is feasible. To this end, we added each of the two biotinylated SUMO-1 probes to whole cell extracts of HEK293T cells that were transiently transfected to express HA-DPP9. After photo-crosslinking, enrichment and stringent washing, we performed an on-bead tryptic digest followed by LC-MS/MS analysis and label-free quantification (LFQ) (Figure 4A). We refer to the sample treated this way as the UV(+) sample. To determine SUMO-1 binders that survived the harsh washing conditions without being crosslinked, a sample without UV-irradiation was prepared and similarly analyzed by LC-MS/MS (UV(-) sample). In addition, proteins that bound non-specifically to the beads were determined in a bead-control lacking a SUMO-1 probe. These latter proteins as well as common contaminations (Cox et al., 2014) and proteins not being identified with a minimum of 2 peptides in 3 out of the 3 biological replicates were manually removed from the list of potential SUMO-1 binders. To determine significantly enriched SUMO-1 binders for each probe, we applied a Student’s t-test (s0 = 0.5 and FDR <0.01). We found HA-DPP9 to be significantly enriched by SUMO-1 (Q69BzF) after UV-irradiation compared to SUMO-1 (R54BzF) (Figure 4B, Supplementary Table S1), consistent with our above-described biochemical probe characterization. HA-DPP9 was neither detected in the UV(-) sample nor in the bead-control, showing that the enrichment was dependent on photo-crosslink-formation.

We then turned to identify interactors of both SUMO-1 (Q69BzF) and SUMO-1 (R54BzF) from the whole proteome. To this end, we used cell extracts of untransfected HeLa cells, which are the standard cell line for such experiments. Using the same workflow, we prepared UV(+) and UV(-) samples as well as bead control samples and analyzed them by LC-MS/MS. As expected, only few proteins survived the harsh washing conditions and were determined as non-covalent SUMO binders for SUMO-1 (Q69BzF) and SUMO-1 (R54BzF) in UV(-) samples, mainly known SUMO interactors like RanBP2, Ubc9 and UBA2 (Supplementary Table S2). UV(+) samples were quantified by LFQ, which resulted in a final list of 644 identified proteins as SUMO-1 binders. By applying a Student’s t-test (s0 = 0.5 and FDR<0.01) we revealed that 192 proteins of these were significantly enriched by SUMO-1 (Q69BzF), whereas 96 proteins were determined as preferred SUMO-1 (R54BzF) binders (Figure 4C, Supplementary Table S2).

Again, in keeping with our probe design, we found known SIM-mediated binders like EXOSC9, CDK2, UBA2, MTA1 and PIAS2 to be enriched by the SUMO-1 (R54BzF) probe. Likewise, we found DPP9 and Ubc9 significantly enriched by the SUMO-1 (Q69BzF) probe. These findings underline the utility of our approach to specifically identify new class II interactors either being direct SUMO-1 binders equipped with a SUMO interaction site or indirect interactors, as part of SUMO-binding protein complexes that mediated sufficient spatial proximity for crosslinking.

**FIGURE 5:**
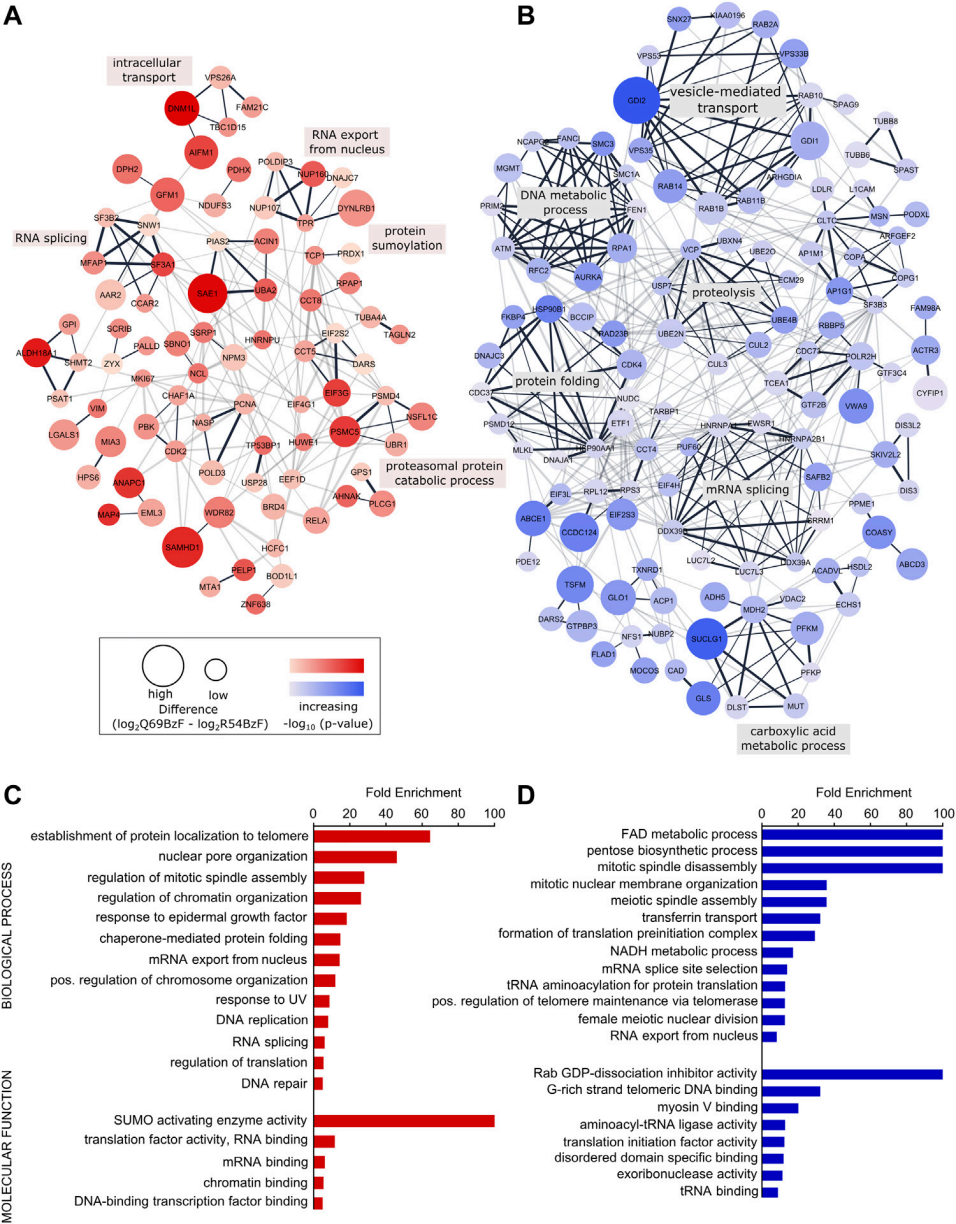
Analysis of identified SUMO-1 (R54BzF) and SUMO-1 (Q69BzF) interactors. **(A)** and **(B)** STRING network analysis and MCL clustering of proteins significantly enriched by SUMO-1 (R54BzF) (red) and SUMO-1 (Q69BzF) (blue) probes, respectively. Nodes are sized according to differences determined by Student’s t-test, which correspond to enrichment compared to the respective other SUMO-1 probe. Color intensities represents–log10 (*p*-value). Only connected nodes are visualized. In the chase of the SUMO-1 (Q69BzF) probe only clusters with three or more proteins are visualized. Edge thickness corresponds to combined scores from STRING. MCL cluster edges are presented in black. Most significant biological process were annotated for large MCL clusters (see also Supplementary Table S3). **(C)** and **(D)** Overrepresentation analysis of Gene Ontology (GO) terms of biological processes and molecular functions compared to the whole human genome, as derived from the SUMO-1 (R54BzF) (red) and SUMO-1 (Q69BzF) (blue) data sets, respectively. Using PANTHER with Fisher’s Exact test only GO terms with FDR *p*-value < 0.05 were considered as significant. For complete list, see Supplementary Table S4.

To gain further insight into the interactors enriched by each SUMO probe, we created physical and functional associated networks using STRING and determined tightly connected protein clusters using the Markov Clustering Algorithm (MCL) (Morris et al., 2011; Szklarczyk et al., 2021). Protein clusters associated with typical SUMO-related biological processes such as RNA processing, DNA metabolic processes as well as protein localization and transport were determined for SUMO-1 probes (Figures 5A,B, Supplementary Table S3). For example, R54BzF binders resulted in a cluster related to protein sumoylation, whereas proteins significantly enriched by Q69BzF formed a large cluster linked to vesicle-mediated transport. To determine more specific gene ontologies (GO terms) associated to biological processes and molecular functions that were statistically overrepresented in each SUMO-1 probe interactor list compared to the whole human genome, we performed a PANTHER analysis (Mi et al., 2021). In agreement with Clustering, GO terms connected to protein localization to telomere and nuclear pore organization were enriched by the SUMO-1 (R54BzF) probe, whilst different small molecule metabolic processes as well as spindle organization are examples for statistically overrepresentated biological processes in the SUMO-1 (Q69BzF) protein list (Figures 5C,D, Supplementary Table S4).

A comparison of our results with other major SUMO proteomic studies (Aguilar-Martinez et al., 2015; Cox et al., 2017; González-Prieto et al., 2021) further validates the feasibility of our photo-crosslinker approach to enrich SUMO binders (see Supplementary Figure S5 for comparison). Importantly, our probes targeted against class I and class II binders can provide valuable insights into SUMO binding modes of newly and previously identified SUMO binders. We determined that 32 SUMO binders previously identified in a SUMO paralog independent studies using non-covalent pulldowns or microarrays (Aguilar-Martinez et al., 2015; Cox et al., 2017; González-Prieto et al., 2021) can be categorized as preferred class II interactors based on their enrichment by the SUMO-1 (Q69BzF) compared to the SUMO-1 (R54BzF) probe (see Supplementary Figure S5B, Supplementary Table S5). We therefore assume that these proteins, among others, could be interesting candidates for further characterization of the class II binding properties.

Finally, also the remaining members of the 356 proteins identified in this study, which did not pass the significance threshold for relative enrichment of class I or class II binders, are of great interest. 80% of these proteins were not identified so far as SUMO binders in other proteomic studies (Supplementary Figure S5C). We assume that weak and transient binding is one explanation for their lacking discovery up to now, as such proteins can be captured with our approach and 343 of these were only determined after covalent crosslinking. Their lacking enrichment for one of our probes may indicate physical contact to both class I and class II interaction surfaces of SUMO-1, or spatial proximity in multiple orientations in case of non-direct binders. Given that they were captured by both the class I and class II crosslinking probes, we believe they represent direct or indirect SUMO-1 interactors of great certainty and should thus also be interesting candidates for future studies.

## Conclusion

Up to this study, only few proteins were shown to interact with the second SUMO-1 binding site that includes E67. We have established a novel SUMO-1 probe capable of specific photo-crosslinking class II SUMO-1 interactors as recombinant proteins and in mammalian cell extracts. By strategic positioning of the photo-crosslinking unnatural amino acid we were able to distinguish proteins which preferentially bind to the SIM binding groove of SUMO-1 (class I) or to the opposite site where the E67 interaction loop (class II) is located. 192 and 96 proteins specifically enriched with either of these two probes, respectively, solidifying the relevance of E67 interaction loop for protein-protein interactions. This study provides new insights into binding preferences of SUMO-1 interactors and are expected to represent interesting candidates for further studies.

## Materials and Methods

### Protein Expression and Purification

Recombinant proteins, with the exception of DPP9, were produced in *E. coli* BL21 (DE3) Gold cells after transformation of the respective plasmid (see Supplementary Table S6 and Supplementary Table S7). An additional plasmid encoding for the orthogonal BzFRS/BzFtRNA_CUA_ pair derived from *M. jannaschii* (Chin et al., 2002) was co-transformed for BzF incorporation. Lysogeny broth (LB) was used for culturing cells and protein expression of the MBP-containing construct as well as Ubc9 was induced at an OD_600_ of 0.8 with 0.4 mM IPTG and performed for 4 h at 28 and 37°C, respectively. SUMO-1 construct expression was induced at an OD_600_ of 0.6–0.8 with 1 mM IPTG and 0.02% arabinose after the addition of 1 mM BzF (dissolved in 1 M NaOH) and was conducted for 4 h at 37°C. Cells were lysed using an emulsifier (Avestin EmulsiFlex®-C5 high pressure emulsifier) followed by centrifugation (12,000 rpm, 30 min, 4°C) and protein purification *via* Ni-NTA affinity chromatography using a gravity flow column with Ni-NTA resins (Cube Biotech). Proteins were eluted with 250 mM Imidazol and dialyzed against TB buffer (20 mM HEPES, 110 mM KOAc, 2 mM MgOAc, 1 mM EGTA, pH 7.3) + 10% glycerol. Further purification of SUMO-1 constructs was performed by size exclusion chromatography (Äkta Purifier [GE Healthcare], Superdex 75 column) using a flow rate of 1 ml/min. For Ubc9 purification, a protocol previously published (Werner et al., 2009) was applied and the protein was dialyzed against TB buffer (pH 7.3) + 10% glycerol. Expression of His_6_-TEV-DPP9-short in SF9 insect cells and purification was performed as described previously (Pilla et al., 2012) (see Supplementary Table S8).

### Bioconjugation of SUMO-1 Probes With Biotin-Maleimide

Bioconjugation of SUMO-1 (80 µM) with 5 eq. Biotin C2 maleimide (ATT Bioquest, dissolved in DMSO) was performed in Tris buffer (50 mM Tris, 300 mM NaCl; pH 7.0) after 15 min reduction with Tris (2-carboxyethyl)phosphine (TCEP, 120 µM) at room temperature. After 1 h incubation, the unreacted Biotin was separated by Ni-NTA affinity chromatography followed by dialysis against PBS buffer (pH 7.4).

### Cell Culture and Transfection

HEK293T cells were cultured in MEM Eagle supplemented with 10% fetal calf serum, 1% penicillin/streptomycin, 1% non-essential amino acids and 1% L-glutamine at 37°C and 5% CO_2_. Cells were transiently transfected using calciumphosphate precipitation method (see Supplementary Table S9 for plasmid information). After 3 h incubation at 37°C and 5% CO_2_, glycerol shock was performed at room temperature for 2 min. Cells were harvested after 48 h by centrifugation (3,500 rpm, 3 min, 4°C) and resuspended in TB buffer +1 mM DTT (supplemented with HALT protease inhibitor cocktail, Thermo Scientific). Lysis for western blot analyses was performed by passing cell suspension through a 26G needle followed by centrifugation (30 min, 14,000 rpm, 4°C). Lysis of cells for proteomic experiments were performed by sonication (10 s pulse, 10 s pause, 30%, 8 min, Bandelin Sonopuls UW3100), followed by centrifugation and dialysis against PBS buffer (pH 7.4) + 1 mM DTT and PBS buffer (pH 7.4) + 20% glycerol. Protein concentrations were determined by BCA assay.

### Preparation of HeLa Whole Cell Extract

HeLa S3 cells were cultured in suspension in MEM (supplemented with 10% fetal calf serum, 1% penicillin/streptomycin, 1% non-essential amino acids, 1% L-glutamine and 1% sodium pyruvate) at 37°C and 5% CO_2_. Cells were harvested by centrifugation and lysed by sonication (10 s pulse, 10 s pause, 30%, 8 min, Bandelin Sonopuls UW3100). After centrifugation, the cell lysate was dialysed against PBS buffer (pH 7.4) + 1 mM DTT and PBS buffer (pH 7.4) + 20% glycerol. Protein concentrations were determined by BCA assay.

### Photo-Induced Crosslinking Assay

SUMO-1 protein (20 µM) was incubated for 15 min with respective binding partner (10 μM; DPP9: 1.5 µM) or HEK293T cell lysate (2.5 mg/ml) at 4°C. Crosslinking was performed in 0.2 ml thin-walled polypropylene PCR tubes (Greiner Bio One) for 60 min using long-wave UV light ( λ=365 nm , Herolab UV-16L, 8 W, 6 mm distance). Before and after UV-irradiation, samples were taken for SDS-PAGE (Coomassie stained) and Western Blotting. The primary antibodies anti-HA (sc-7392) and anti-His (T505) together with HRP-conjugated secondary antibody was used. Chemiluminescence detection was performed by ECL Western Blotting Analysis system (GE Healthcare).

Crosslink bands visualized by SDS-PAGE and Coomassie-staining were further quantified by densitometric analysis of the band intensities using the software Lazer, 2000 (Dr. Istvan Lazar, www.gelanalyzer.com). The band intensities were normalized accourding to their molecular weight and the crosslink intensity was subsequently calculated as the proportion (in %) of crosslink product to the sum of unreacted and reacted SUMO binding partner. The mean and standard deviation was determined from three replicates.

### Enrichment of SUMO-1 Binding Partners From Cell Extracts

Biotinylated SUMO-1 probe (15 µM) was mixed with HEK293T whole cell extract of cells previously transfected with HA-DPP9 or HeLa S3 whole cell extract (8 mg/ml, supplemented with HALT protease inhibitor cocktail, Thermo Scientific) and incubated for 15 min at 4°C. Half of the sample was UV-irradiated for 1 h ( λ=365 nm , Herolab UV-16L, 8 W, 6 mm distance) at room temperature [termed UV(+)]. The other half was incubated without UV irradiation for 1 h at RT (termed UV(-)). Similarly, “bead control” samples were prepared without a SUMO-1 probe. After incubation with streptavidin Sepharose beads (GE Healthcare, 17-5,113–01) for 2 h at 4°C, a stringent washing protocol (Tagwerker et al., 2006) was applied using spin columns (Pierce Micro-Spin Columns, 10510824) and the following buffers: buffer A (8 M urea, 2% SDS, 100 mM Tris, 200 mM NaCl, pH 8), buffer B (8 M urea, 0.2% SDS, 10% ethanol, 10% isopropanol, 100 mM Tris, 1.2 M NaCl, pH 8), buffer C and D (8 M Urea, 0.2% SDS, 10% ethanol, 10% isopropanol, 100 mM Tris, 200 mM NaCl, pH5 and pH 9), buffer E (100 mM Tris, 200 mM NaCl, pH 8), and buffer F (50 mM NH_4_CO_3_). An on-bead tryptic digest was performed after reduction (5 mM DTT, 30 min, 56°C), alkylation [25 mM 2-iodoacetamide (IAA), 20 min, RT] and quenching (15 mM DTT) through the addition of 200 ng trypsin (dissolved in 50 mM NH_4_HCO_3_) and ProteaseMax (Promega Corp.). After overnight incubation at 37°C in a rotation wheel, the supernatant was vacuum-dried, resuspended in acetonitrile/water (2% v/v), acidified with formic acid (0.1% v/v) and analysed by LC-MS/MS.

### In-Gel Digest of Purified Proteins After Photo-Induced Crosslinking

In-gel digest was performed as previously described (Brüninghoff et al., 2020). In short, the excised gel band was destained (50% methanol, 0.1% TFA in water, 40°C) followed by acetonitrile treatment and vacuum-drying in a speedvac. The samples were reduced by DTT (10 mM in 100 mM NH_4_HCO_3_, 20 min, 56°C) and alkylated (55 mM IAA in 100 mM NH_4_HCO_3_, 30 min, RT). 200 ng trypsin in 50 mM NH_4_HCO_3_ and ProteaseMAX was added and the samples were incubated for 2 h at 37°C. After vacuum-drying using a speedvac, the samples were resuspended in 2% acetonitrile/0.1% formic acid and analysed by LC-MS/MS.

### LC-MS/MS

LC-MS/MS analysis was performed using an UltiMate™ 3000 RS LC nano system (Thermo Fisher Scientific GmbH, Dreieich, Germany) connected to a maXis II UHR-qTOF mass spectrometer with a nano-ESI source (CaptiveSpray with nanoBooster, Bruker Daltonik GmbH, Bremen, Germany). The sample [in-gel digest: 5 μL; proteomic sample (on bead digest): 1 µL] was loaded and desalted for 20 min on a C18 trapping column (Thermo Acclaim PepMap100, C18, 5 μm, 0.1 mm × 20 mm, Thermo Fisher Scientific GmbH, Dreieich, Germany) at a flow rate of 5 μL/min in 2% eluent B (eluent A: 0.1% formic acid in water; eluent B: 0.1% formic acid in acetonitrile). During this 20 min period, a sodium formate solution (0.5 mM in 50% 2-propanol) was injected for subsequent internal calibration of the resulting data files. The in-gel digest sample was analyzed using a 50 min gradient (2%–60% B, flow rate 300 nL/min) for the separation on a C18 nano column (Acclaim™ PepMap™ 100 C18, 2 μm, 100 Å, ID 0.075 mm × L 250 mm, Thermo Fisher Scientific GmbH, Dreieich, Germany). The proteomic sample (on-bead digest) was separated on a C18 nano column applying a 150 min gradient (2%–42% B, flow rate 300 nL/min). MS settings: capillary voltage 1.400 V, mass range: m/z 150-2200. The UV(+) proteomic samples were measured with four technical replicates. The bead control and UV(-) proteomic samples were measured with two to three technical replicates. MS survey scans were performed with a cycle time of 5 s. After each survey scan, the 20–30 most abundant precursor ions with z > 1 were selected for fragmentation using collision-induced dissociation. The MS/MS spectra rate was adjusted depending on the precursor intensity. The precursor isolation window and the collision energy were depending on the precursor m/z and charge. DataAnalysis 4.4 (Bruker Daltonik GmbH, Bremen, Germany) was used for chromatogram processing. MSMS peak lists of deconvoluted fragment ion peaks where exported as mgf files and crosslinked peptides were identified using Stavrox 3.6 with the following settings: 2 missed cleavages were allowed; carbamidomethylation of Cys was set as static modification and up to 3 methionine oxidations were set as variable modification. All proteinogenic amino acids and oxidized methionine were considered as potential reaction sites for the crosslinker BzF. Precursor precision: 10 ppm; Fragment ion precision: 10 ppm. Crosslink candidates with a score greater than 100 were considered. (Götze et al., 2012).

### Raw Data Processing

Raw files of proteomic samples were analysed using MaxQuant software v1.6.17 (Cox and Mann, 2008) and Uniprot complete human proteome (UP000005640, downloaded at 19.11.2021). Oxidation and N-terminal acetylation were allowed as variable modification and Carbamidomethylation was set as fixed modification with a maximum number of 5 modifications per peptide. Trypsin/P digestion was selected with a maximum of 2 missed cleavages. Match between runs was enabled with a match time window of 0.7 min and an alignment time window of 20 min. For the analysis of UV(+) samples, label-free quantification (LFQ) with an LFQ minimum ratio count of 2 was performed (Cox et al., 2014). Fast LFQ was disabled. Unique and razor peptides were considered for quantification.

### Data Analysis

The MaxQuant output file “proteingroup.txt” of UV(+) analysis was further processed using Perseus (v1.6.15) (Tyanova et al., 2016). Proteins only identified by modified peptides as well as contaminants and reverse hits were removed. LFQ intensities were log2 transformed and the biological replicates of each SUMO-1 probe were grouped accordingly. The matrix was filtered for proteins with 3 out of 3 (HEK293T cell extract) or 6 out of 6 (HeLa cell extract) valid values in at least one group. Valid values were defined as LFQ intensities >0 as well as a minimum number of 2 peptides per protein. Missing values were imputed from a normal distribution with a width of 0.3 and a down shift of 1.8 taking into account the total matrix. Student’s t-test was performed with S0 = 0.5 and a permutation-based FDR<0.01 (number of randomization = 250). Volcano plots were visualized with Graphpad Prism 6 using the respective cut-off curves exported from Perseus.

MaxQuant output files of UV(-) and bead-controls were further processed in MS Excel. For the experiment using HEK293T cell extracts with overexpressed DPP9, proteins considered as identified in UV(-) for each SUMO-1 probe had to be determined with at least 2 peptides in 3 of 3 biological replicates and are listed in Supplementary Table S1. Proteins in bead-control samples were considered as identified if at least 2 peptides were determined in 2 out of 3 biological replicates. These proteins were excluded from the respective UV(-) and UV(+) lists. For the experiment using HeLa whole cell extract, proteins considered as SUMO-1 binders in the respective UV(-) samples had to be identified with at least 2 peptides in 6 out of 6 biological replicates and are listed in Supplementary Table S2. Proteins in bead-control samples were regarded as identified when at least 2 peptides were present in 4 out of 6 biological replicates. These proteins were excluded from the respective UV(-) and UV(+) lists.

### Bioinformatic Analysis

Full STRING networks were generated for proteins specifically enriched by SUMO-1 (R54BzF) and SUMO-1 (Q69BzF) probe, respectively, using STRING database (v11.5, https://string-db.org/) and were further analyzed with Cytoscape (v3.8.2) (Shannon et al., 2003; Szklarczyk et al., 2021). Interactions with a medium confidence score (0.4) were considered and only connected nodes were visualized. Clustering was performed in Cytoscape using MCL algorithm with an inflation of 3. Most enriched biological process of clusters were determined by STRING Enrichment App in Cytoscape (see Supplementary Table S3) (Morris et al., 2011; Doncheva et al., 2019).

PANTHER was used to determine gene ontologies statistically overrepresented in the protein lists enriched by the respective SUMO-1 probes compared to the whole human genome (Mi et al., 2021). Fisher’s Exact test was applied and GO terms with FDR *p*-value < 0.05 were regarded as significant. Enriched GO terms associated with biological processes and molecular functions are listed in Supplementary Table S4.

## Data Availability

The mass spectrometry proteomics data have been deposited to the ProteomeXchange Consortium via the PRIDE partner repository (Perez-Riverol et al., 2022) with the dataset identifier PXD033107.

## References

[B1] Aguilar-Martinez E. Chen X. Webber A. Mould A. P. Seifert A. Hay R. T. (2015). Screen for Multi-SUMO-Binding Proteins Reveals a Multi-SIM-Binding Mechanism for Recruitment of the Transcriptional Regulator ZMYM2 to Chromatin. Proc. Natl. Acad. Sci. U.S.A. 112, E4854–E4863. 10.1073/pnas.1509716112 26283374 PMC4568223

[B2] Baba D. Maita N. Jee J.-G. Uchimura Y. Saitoh H. Sugasawa K. (2005). Crystal Structure of Thymine DNA Glycosylase Conjugated to SUMO-1. Nature 435, 979–982. 10.1038/nature03634 15959518

[B3] Belsom A. Mudd G. Giese S. Auer M. Rappsilber J. (2017). Complementary Benzophenone Cross-Linking/Mass Spectrometry Photochemistry. Anal. Chem. 89, 5319–5324. 10.1021/acs.analchem.6b04938 28430416 PMC5441754

[B4] Brantis-de-Carvalho C. E. Maarifi G. Gonçalves Boldrin P. E. Zanelli C. F. Nisole S. Chelbi-Alix M. K. (2015). MxA Interacts with and Is Modified by the SUMOylation Machinery. Exp. Cell Res. 330, 151–163. 10.1016/j.yexcr.2014.10.020 25447205

[B5] Brüninghoff K. Aust A. Taupitz K. F. Wulff S. Dörner W. Mootz H. D. (2020). Identification of SUMO Binding Proteins Enriched after Covalent Photo-Cross-Linking. ACS Chem. Biol. 15, 2406–2414. 10.1021/acschembio.0c00609 32786267

[B6] Capili A. D. Lima C. D. (2007). Structure and Analysis of a Complex between SUMO and Ubc9 Illustrates Features of a Conserved E2-Ubl Interaction. J. Mol. Biol. 369, 608–618. 10.1016/j.jmb.2007.04.006 17466333 PMC1940065

[B7] Chang H.-M. Yeh E. T. H. (2020). SUMO: From Bench to Bedside. Physiol. Rev. 100, 1599–1619. 10.1152/physrev.00025.2019 32666886 PMC7717128

[B8] Chin J. W. Martin A. B. King D. S. Wang L. Schultz P. G. (2002). Addition of a Photocrosslinking Amino Acid to the Genetic Code of *Escherichia coli* . Proc. Natl. Acad. Sci. U.S.A. 99, 11020–11024. 10.1073/pnas.172226299 12154230 PMC123203

[B9] Cox E. Hwang W. Uzoma I. Hu J. Guzzo C. M. Jeong J. (2017). Global Analysis of SUMO-Binding Proteins Identifies SUMOylation as a Key Regulator of the INO80 Chromatin Remodeling Complex. Mol. Cell. Proteomics 16, 812–823. 10.1074/mcp.M116.063719 28254775 PMC5417823

[B10] Cox J. Hein M. Y. Luber C. A. Paron I. Nagaraj N. Mann M. (2014). Accurate Proteome-wide Label-free Quantification by Delayed Normalization and Maximal Peptide Ratio Extraction, Termed MaxLFQ. Mol. Cell. Proteomics 13, 2513–2526. 10.1074/mcp.M113.031591 24942700 PMC4159666

[B11] Cox J. Mann M. (2008). MaxQuant Enables High Peptide Identification Rates, Individualized p.p.b.-range Mass Accuracies and Proteome-wide Protein Quantification. Nat. Biotechnol. 26, 1367–1372. 10.1038/nbt.1511 19029910

[B12] Danielsen J. R. Povlsen L. K. Villumsen B. H. Streicher W. Nilsson J. Wikström M. (2012). DNA Damage-Inducible SUMOylation of HERC2 Promotes RNF8 Binding via a Novel SUMO-Binding Zinc Finger. J. Cell Biol. 197, 179–187. 10.1083/jcb.201106152 22508508 PMC3328386

[B13] Diehl C. Akke M. Bekker-Jensen S. Mailand N. Streicher W. Wikström M. (2016). Structural Analysis of a Complex between Small Ubiquitin-like Modifier 1 (SUMO1) and the ZZ Domain of CREB-Binding Protein (CBP/p300) Reveals a New Interaction Surface on SUMO. J. Biol. Chem. 291, 12658–12672. 10.1074/jbc.M115.711325 27129204 PMC4933466

[B14] Doncheva N. T. Morris J. H. Gorodkin J. Jensen L. J. (2019). Cytoscape StringApp: Network Analysis and Visualization of Proteomics Data. J. Proteome Res. 18, 623–632. 10.1021/acs.jproteome.8b00702 30450911 PMC6800166

[B15] Dormán G. Nakamura H. Pulsipher A. Prestwich G. D. (2016). The Life of Pi Star: Exploring the Exciting and Forbidden Worlds of the Benzophenone Photophore. Chem. Rev. 116, 15284–15398. 10.1021/acs.chemrev.6b00342 27983805

[B16] Duda D. M. van Waardenburg R. C. A. M. Borg L. A. McGarity S. Nourse A. Waddell M. B. (2007). Structure of a SUMO-Binding-Motif Mimic Bound to Smt3p-Ubc9p: Conservation of a Non-covalent Ubiquitin-like Protein-E2 Complex as a Platform for Selective Interactions within a SUMO Pathway. J. Mol. Biol. 369, 619–630. 10.1016/j.jmb.2007.04.007 17475278 PMC1936411

[B17] Flotho A. Melchior F. (2013). Sumoylation: a Regulatory Protein Modification in Health and Disease. Annu. Rev. Biochem. 82, 357–385. 10.1146/annurev-biochem-061909-093311 23746258

[B18] Geiss-Friedlander R. Melchior F. (2007). Concepts in Sumoylation: a Decade on. Nat. Rev. Mol. Cell Biol. 8, 947–956. 10.1038/nrm2293 18000527

[B19] González-Prieto R. Eifler-Olivi K. Claessens L. A. Willemstein E. Xiao Z. Talavera Ormeno C. M. P. (2021). Global Non-Covalent SUMO Interaction Networks Reveal SUMO-dependent Stabilization of the Non-homologous End Joining Complex. Cell Rep. 34, 108691. 10.1016/j.celrep.2021.108691 33503430

[B20] Götze M. Pettelkau J. Schaks S. Bosse K. Ihling C. H. Krauth F. (2012). StavroX-A Software for Analyzing Crosslinked Products in Protein Interaction Studies. J. Am. Soc. Mass Spectrom. 23, 76–87. 10.1007/s13361-011-0261-2 22038510

[B21] Hannich J. T. Lewis A. Kroetz M. B. Li S.-J. Heide H. Emili A. (2005). Defining the SUMO-Modified Proteome by Multiple Approaches in *Saccharomyces cerevisiae**. J. Biol. Chem. 280, 4102–4110. 10.1074/jbc.M413209200 15590687

[B22] Hay R. T. (2013). Decoding the SUMO Signal. Biochem. Soc. Trans. 41, 463–473. 10.1042/BST20130015 23514139

[B23] Hecker C.-M. Rabiller M. Haglund K. Bayer P. Dikic I. (2006). Specification of SUMO1- and SUMO2-Interacting Motifs. J. Biol. Chem. 281, 16117–16127. 10.1074/jbc.M512757200 16524884

[B24] Hendriks I. A. Vertegaal A. C. O. (2016). A Comprehensive Compilation of SUMO Proteomics. Nat. Rev. Mol. Cell Biol. 17, 581–595. 10.1038/nrm.2016.81 27435506

[B25] Kerscher O. (2007). SUMO Junction-What's Your Function? New Insights through SUMO-Interacting Motifs. EMBO Rep. 8, 550–555. 10.1038/sj.embor.7400980 17545995 PMC2002525

[B26] Keusekotten K. Bade V. N. Meyer-Teschendorf K. Sriramachandran A. M. Fischer-Schrader K. Krause A. (2014). Multivalent Interactions of the SUMO-Interaction Motifs in RING Finger Protein 4 Determine the Specificity for Chains of the SUMO. Biochem. J. 457, 207–214. 10.1042/BJ20130753 24151981 PMC3901395

[B27] Knipscheer P. van Dijk W. J. Olsen J. V. Mann M. Sixma T. K. (2007). Noncovalent Interaction between Ubc9 and SUMO Promotes SUMO Chain Formation. EMBO J. 26, 2797–2807. 10.1038/sj.emboj.7601711 17491593 PMC1888673

[B28] Kötter A. Mootz H. D. Heuer A. (2019). Standard Binding Free Energy of a SIM-SUMO Complex. J. Chem. Theory Comput. 15, 6403–6410. 10.1021/acs.jctc.9b00428 31525924

[B52] Lazer I. (2000). Gel Analyzer 2010a:Freeware 1D gel electrophoresis image analysis software. Available at: http://www.gelanalyzer.com

[B29] Liu C. C. Schultz P. G. (2010). Adding New Chemistries to the Genetic Code. Annu. Rev. Biochem. 79, 413–444. 10.1146/annurev.biochem.052308.105824 20307192

[B30] Liu Q. Jin C. Liao X. Shen Z. Chen D. J. Chen Y. (1999). The Binding Interface between an E2 (UBC9) and a Ubiquitin Homologue (UBL1). J. Biol. Chem. 274, 16979–16987. 10.1074/jbc.274.24.16979 10358047

[B31] Mi H. Ebert D. Muruganujan A. Mills C. Albou L.-P. Mushayamaha T. (2021). PANTHER Version 16: a Revised Family Classification, Tree-Based Classification Tool, Enhancer Regions and Extensive API. Nucleic acids Res. 49, D394–D403. 10.1093/nar/gkaa1106 33290554 PMC7778891

[B32] Mishra P. K. Yoo C. M. Hong E. Rhee H. W. (2020). Photo-crosslinking: An Emerging Chemical Tool for Investigating Molecular Networks in Live Cells. Chembiochem 21, 924–932. 10.1002/cbic.201900600 31794116

[B33] Morris J. H. Apeltsin L. Newman A. M. Baumbach J. Wittkop T. Su G. (2011). clusterMaker: a Multi-Algorithm Clustering Plugin for Cytoscape. BMC Bioinforma. 12, 436. 10.1186/1471-2105-12-436 PMC3262844 22070249

[B34] Perez-Riverol Y. Bai J. Bandla C. García-Seisdedos D. Hewapathirana S. Kamatchinathan S. (2022). The PRIDE Database Resources in 2022: A Hub for Mass Spectrometry-Based Proteomics Evidences. Nucleic Acids Res. 50, D543–D552. 10.1093/nar/gkab1038 34723319 PMC8728295

[B35] Pichler A. Fatouros C. Lee H. Eisenhardt N. (2017). SUMO Conjugation - a Mechanistic View. Biomol. concepts 8, 13–36. 10.1515/bmc-2016-0030 28284030

[B36] Pilla E. Möller U. Sauer G. Mattiroli F. Melchior F. Geiss-Friedlander R. (2012). A Novel SUMO1-specific Interacting Motif in Dipeptidyl Peptidase 9 (DPP9) that Is Important for Enzymatic Regulation. J. Biol. Chem. 287, 44320–44329. 10.1074/jbc.M112.397224 23152501 PMC3531746

[B37] Praefcke G. J. K. Hofmann K. Dohmen R. J. (2012). SUMO Playing Tag with Ubiquitin. Trends Biochem. Sci. 37, 23–31. 10.1016/j.tibs.2011.09.002 22018829

[B38] Psakhye I. Jentsch S. (2012). Protein Group Modification and Synergy in the SUMO Pathway as Exemplified in DNA Repair. Cell 151, 807–820. 10.1016/j.cell.2012.10.021 23122649

[B39] Saitoh H. Hinchey J. (2000). Functional Heterogeneity of Small Ubiquitin-Related Protein Modifiers SUMO-1 versus SUMO-2/3. J. Biol. Chem. 275, 6252–6258. 10.1074/jbc.275.9.6252 10692421

[B40] Shannon P. Markiel A. Ozier O. Baliga N. S. Wang J. T. Ramage D. (2003). Cytoscape: a Software Environment for Integrated Models of Biomolecular Interaction Networks. Genome Res. 13, 2498–2504. 10.1101/gr.1239303 14597658 PMC403769

[B41] Song J. Durrin L. K. Wilkinson T. A. Krontiris T. G. Chen Y. (2004). Identification of a SUMO-Binding Motif that Recognizes SUMO-Modified Proteins. Proc. Natl. Acad. Sci. U.S.A. 101, 14373–14378. 10.1073/pnas.0403498101 15388847 PMC521952

[B42] Sriramachandran A. M. Meyer-Teschendorf K. Pabst S. Ulrich H. D. Gehring N. H. Hofmann K. (2019). Arkadia/RNF111 Is a SUMO-Targeted Ubiquitin Ligase with Preference for Substrates Marked with SUMO1-Capped SUMO2/3 Chain. Nat. Commun. 10, 3678. 10.1038/s41467-019-11549-3 31417085 PMC6695498

[B43] Szklarczyk D. Gable A. L. Nastou K. C. Lyon D. Kirsch R. Pyysalo S. (2021). The STRING database in 2021: customizable protein-protein networks, and functional characterization of user-uploaded gene/measurement sets. Nucleic Acids Res. 49, D605–D612. 10.1093/nar/gkaa1074 33237311 PMC7779004

[B44] Tagwerker C. Flick K. Cui M. Guerrero C. Dou Y. Auer B. (2006). A Tandem Affinity Tag for Two-step Purification under Fully Denaturing Conditions: Application in Ubiquitin Profiling and Protein Complex Identification Combined with in Vivocross-Linking. Mol. Cell. Proteomics 5, 737–748. 10.1074/mcp.M500368-MCP200 16432255

[B45] Tanaka Y. Bond M. R. Kohler J. J. (2008). Photocrosslinkers Illuminate Interactions in Living Cells. Mol. Biosyst. 4, 473–480. 10.1039/B803218A 18493640

[B46] Taupitz K. F. Dörner W. Mootz H. D. (2017). Covalent Capturing of Transient SUMO-SIM Interactions Using Unnatural Amino Acid Mutagenesis and Photocrosslinking. Chem. Eur. J. 23, 5978–5982. 10.1002/chem.201605619 28121373

[B47] Tyanova S. Temu T. Sinitcyn P. Carlson A. Hein M. Y. Geiger T. (2016). The Perseus Computational Platform for Comprehensive Analysis of (Prote)omics Data. Nat. methods 13, 731–740. 10.1038/nmeth.3901 27348712

[B48] Werner A. Moutty M.-C. Möller U. Melchior F. (2009). Performing *In Vitro* Sumoylation Reactions Using Recombinant Enzymes. Methods Mol. Biol. 497, 187–199. 10.1007/978-1-59745-566-4_12 19107418

[B49] Winnik M. A. (1981). Cyclization and the Conformation of Hydrocarbon Chains. Chem. Rev. 81, 491–524. 10.1021/cr00045a004

[B50] Wittelsberger A. Mierke D. F. Rosenblatt M. (2008). Mapping Ligand-Receptor Interfaces: Approaching the Resolution Limit of Benzophenone-Based Photoaffinity Scanning. Chem. Biol. Drug Des. 71, 380–383. 10.1111/j.1747-0285.2008.00646.x 18312550 PMC2570705

[B51] Wittelsberger A. Thomas B. E. Mierke D. F. Rosenblatt M. (2006). Methionine Acts as a "magnet" in Photoaffinity Crosslinking Experiments. FEBS Lett. 580, 1872–1876. 10.1016/j.febslet.2006.02.050 16516210

