# Supplementary material for: A Photo-Crosslinking Approach to Identify Class II SUMO-1 Binders
Source: Front Chem. 2022 May 30;10:900989. doi: 10.3389/fchem.2022.900989 (PMC9191277; doi:10.3389/fchem.2022.900989)
Supplement: Supplementary file 1 [file DataSheet1.pdf]

SUPPORTING INFORMATION

A Photo-Crosslinking Approach to Identify Class II SUMO-1 Binders

Kira Brüninghoff[1], Stephanie Wulff[1], Wolfgang Dörner[1], Ruth Geiss-Friedlander[2], Henning D. Mootz[1]*

[1]University of Münster, Institute of Biochemistry, Münster, Germany
[2]University of Freiburg, Institute of Molecular Medicine and Cell Research, Freiburg, Germany

Table of contents

Supporting Figures page S2

Supporting Tables page S7

Supporting References page S11

SUPPORTING FIGURES

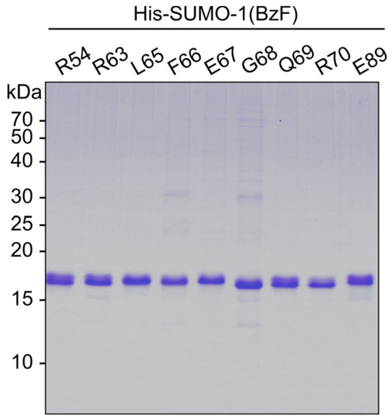

Figure S1. SUMO-1 mutants. SUMO-1 mutants with BzF at different positions were recombinantly expressed in *E.coli* and purified by N-terminal His$_6$-tag using Ni-NTA and size exclusion chromatography.

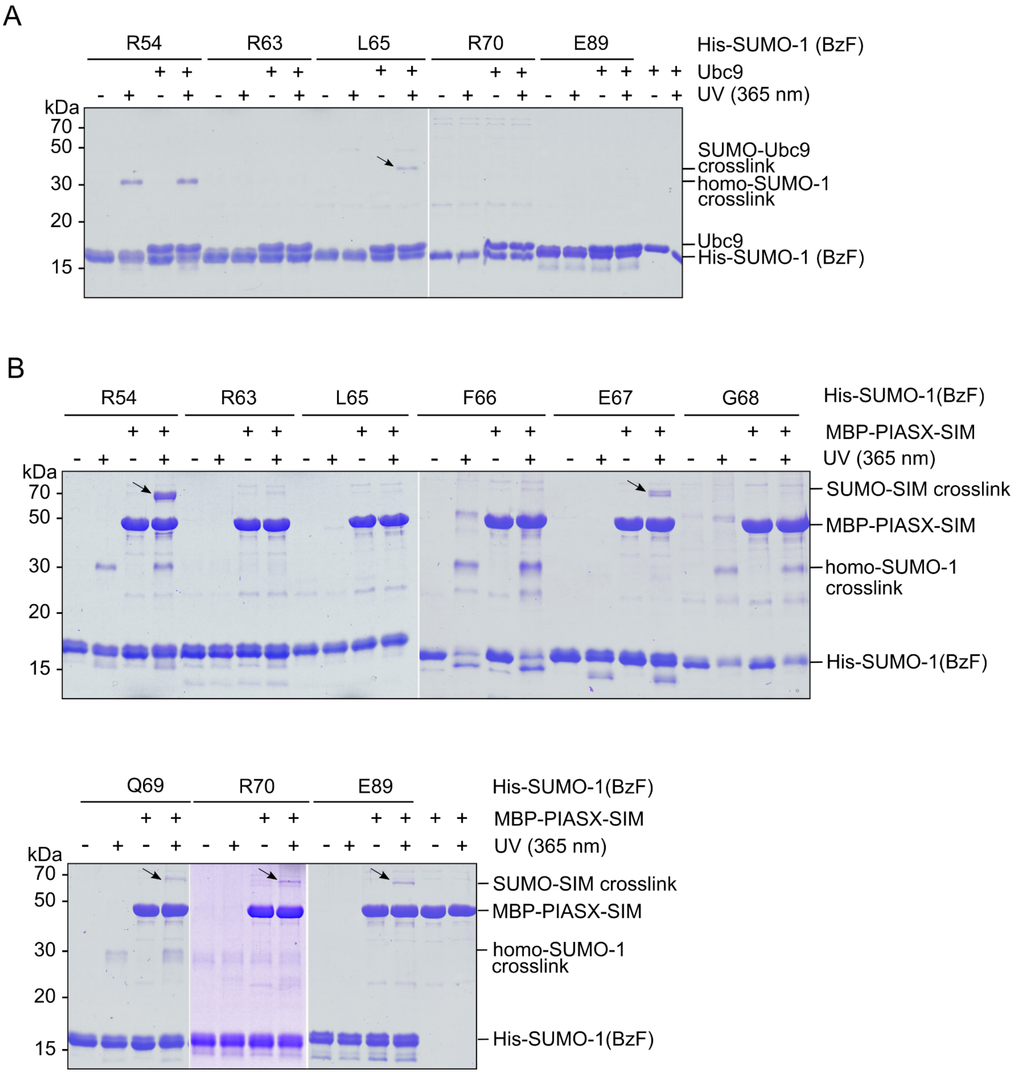

Figure S2. Photo-crosslinking of SUMO-1 mutants with Ubc9 (A) and MBP-PIASX-SIM (B). Proteins were incubated followed by UV-irradiated for 60 min. Samples before and after irradiation were analyzed by SDS-PAGE analysis and Coomassie staining.

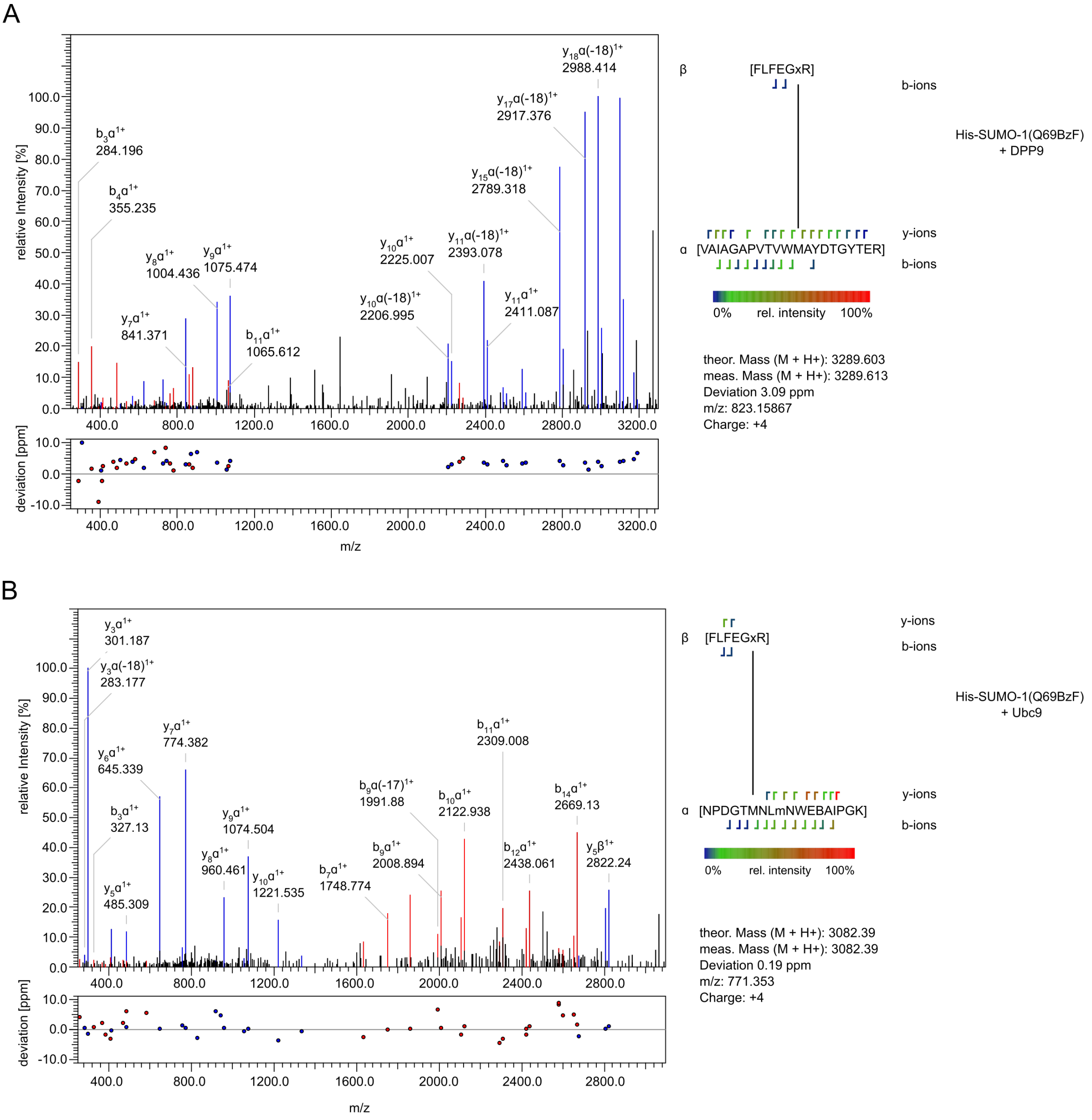

Figure S3. Analyses of photo-crosslinking products of His-SUMO-1(Q69BzF) with class II SUMO-1 binding partners DPP9 and Ubc9. (A) MS/MS spectra and fragmentation representation of the α-β crosslinked product of His-SUMO-1(Q69BzF) (β) and DPP9 (α) peptides. The precursor ion with a charge of +4 was measured at m/z 823.15867. The deviation of the singly charged precursor mass ([M+H$^+$]: 3289.613) from the theoretical singly charged crosslinked product ([M+H$^+$]: 3289.603) is 3.09 ppm. (B) MS/MS spectra and fragmentation representation of the α-β crosslinked product of His-SUMO-1(Q69BzF) (β) and Ubc9 (α) peptides. The precursor ion with a charge of +4 was measured at m/z 771.353. The deviation of the singly charged precursor mass ([M+H$^+$]: 3082.39) from the theoretical singly charged crosslinked product ([M+H$^+$]: 3082.39) is 0.19 ppm. The b- and y-type fragment ions of the peptide backbone are shown as red and blue peaks in the respective MS/MS spectra. Each of the depicted crosslink positions is one of the most likely positions hit by the photo-crosslinker, as can be determined from the accuracy of the data. 'x' represents photo-crosslinker BzF. 'm' indicates oxidized methionine.

A

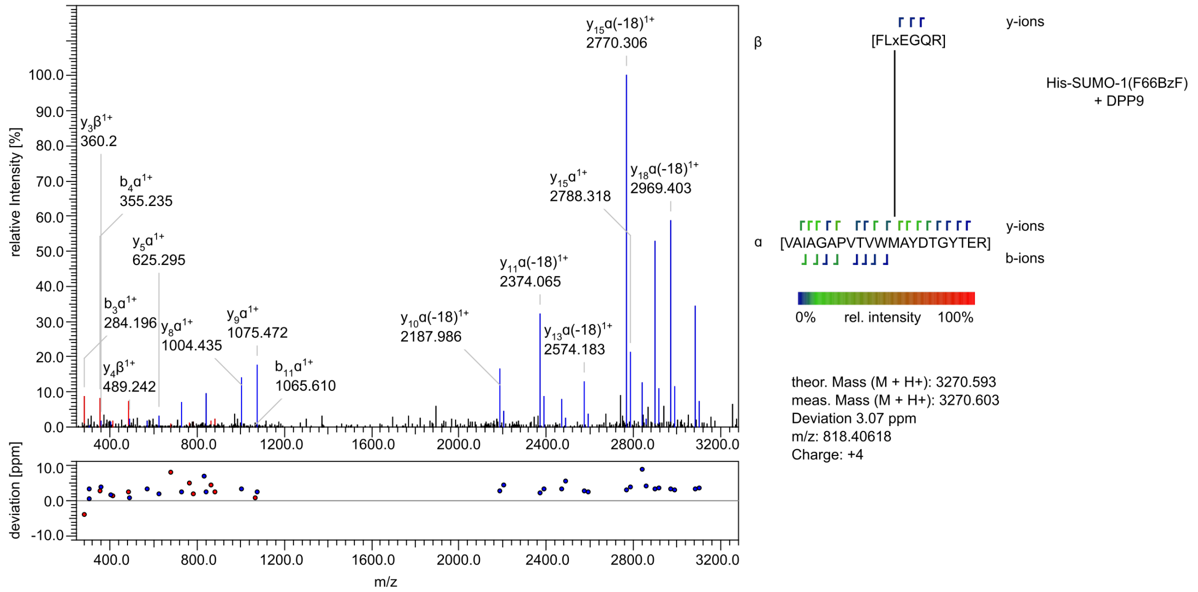

B

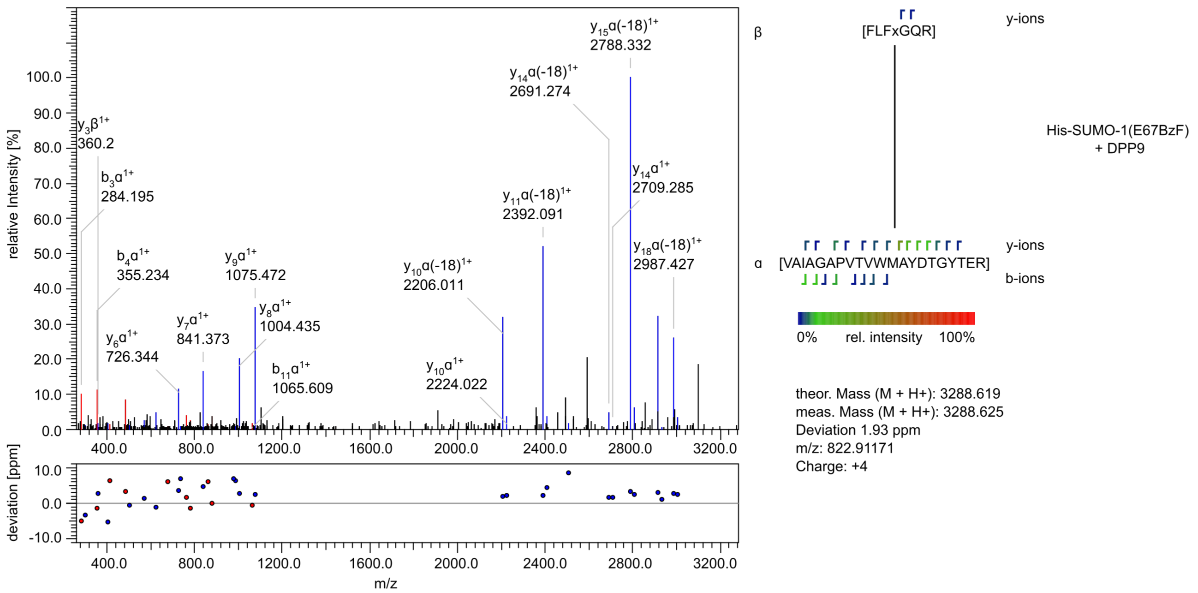

Figure S4. Analyses of photo-crosslinking products of His-SUMO-1(F66BzF) and His-SUMO-1(E67BzF) with DPP9. (A) MS/MS spectra and fragmentation representation of the α-β crosslinked product of His-SUMO-1(F66BzF) (β) and DPP9 (α) peptides. The precursor ion with a charge of +4 was measured at m/z 818.40618. The deviation of the singly charged precursor mass ([M+H$^+$]: 3270.603) from the theoretical singly charged crosslinked product ([M+H$^+$]: 3270.593) is 3.07 ppm. (B) MS/MS spectra and fragmentation representation of the α-β crosslinked product of His-SUMO-1(E67BzF) (β) and DPP9 (α) peptides. The precursor ion with a charge of +4 was measured at m/z 822.91171. The deviation of the singly charged precursor mass ([M+H$^+$]: 3288.625) from the theoretical singly charged crosslinked product ([M+H$^+$]: 3288.619) is 1.93 ppm. Each of the depicted crosslink positions is one of the most likely positions hit by the photo-crosslinker, as can be determined from the accuracy of the data. ‘x’ represents photo-crosslinker BzF.

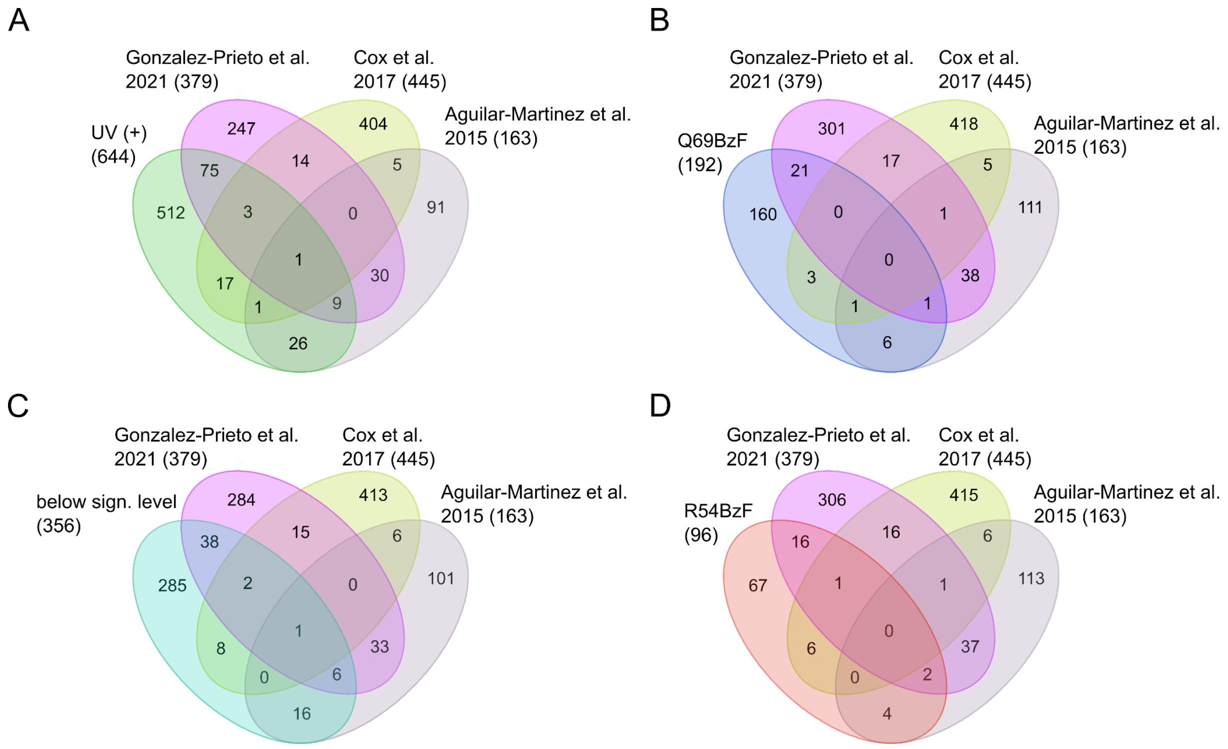

Figure S5. Comparison of all SUMO-binders identified in this study (A), proteins enriched by SUMO-1(Q69BzF) (B), proteins below the significance level (C) and proteins enriched by SUMO-1(R54BzF) (D) with SUMO interactors identified in the recent three major proteomic studies of (González-Prieto et al., 2021), (Cox et al., 2017) and (Aguilar-Martinez et al., 2015). For the comparison analyses, only proteins that could be unambiguously identified with Uniprot were considered.

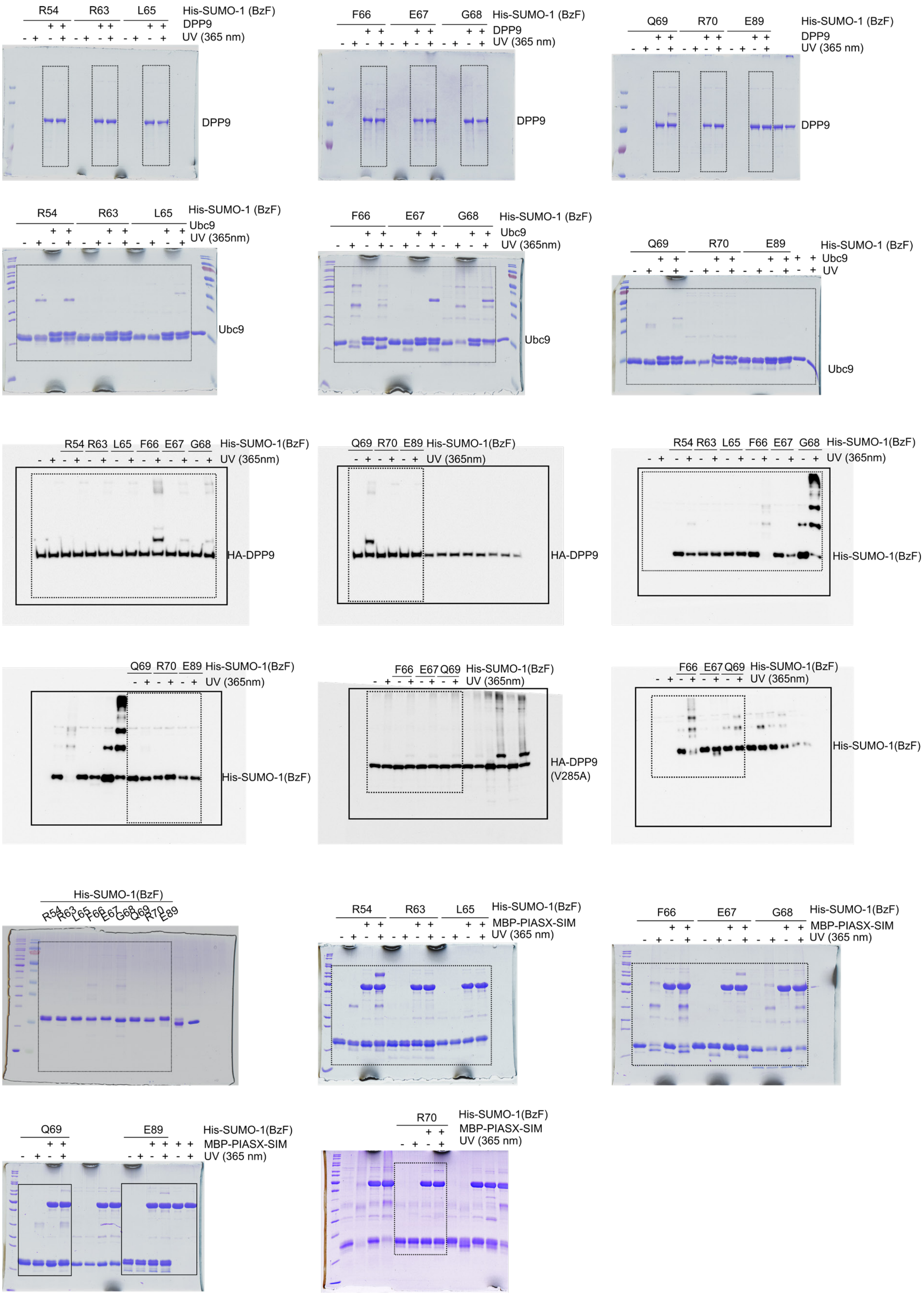

Figure S6. Uncropped images of SDS-gels and blots in Fig. 2, Fig. 3, Fig. S1, Fig. S2.

SUPPORTING TABLES

Tables S1-S5 (Excel sheets) are provided as separate Supporting Information file.

Table S6: List of proteins recombinantly expressed in *E.coli* and their expression plasmids.

**Name of construct**	**Plasmid name**	**Vector backbone**
Met-Gly-Cys-Ser-His$_6$-SUMO-1(C52A, R54BzF)	pKBR42 + pEVOL(BzF)	pET28a
Met-Gly-Cys-Ser-His$_6$-SUMO-1(C52A, R63BzF)	pKBR105 + pEVOL(BzF)	pET28a
Met-Gly-Cys-Ser-His$_6$-SUMO-1(C52A, L65BzF)	pKBR106 + pEVOL(BzF)	pET28a
Met-Gly-Cys-Ser-His$_6$-SUMO-1(C52A, F66BzF)	pKBR111 + pEVOL(BzF)	pET28a
Met-Gly-Cys-Ser-His$_6$-SUMO-1(C52A, E67BzF)	pKBR112 + pEVOL(BzF)	pET28a
Met-Gly-Cys-Ser-His$_6$-SUMO-1(C52A, G68BzF)	pKBR113 + pEVOL(BzF)	pET28a
Met-Gly-Cys-Ser-His$_6$-SUMO-1(C52A, Q69BzF)	pKBR110 + pEVOL(BzF)	pET28a
Met-Gly-Cys-Ser-His$_6$-SUMO-1(C52A, R70BzF)	pKBR107 + pEVOL(BzF)	pET28a
Met-Gly-Cys-Ser-His$_6$-SUMO-1(C52A, E89BzF)	pKBR108 + pEVOL(BzF)	pET28a
Ubc9	pUbc9	pET23a (provided by (Flotho et al., 2012))
MBP-PIASX-SIM-His$_6$	pKT57	pMal-c2X

Table S7: Sequences of proteins used in this study.

**Protein**	**Sequence**
Met-Gly-Cys-Ser-His$_6$-SUMO-1(C52A, R54BzF)	MGCSHHHHHHGSDQEAKPSTEDLGDKKEGEYIKLKVIGQDSSEIHFKVKMTTHLKKLKESYAQ[BzF]QGVPMNSLRFLFEGQRIADNHTPKELGMEEEDVIEVYQEQTGG
Met-Gly-Cys-Ser-His$_6$-SUMO-1(C52A, R63BzF)	MGCSHHHHHHGSDQEAKPSTEDLGDKKEGEYIKLKVIGQDSSEIHFKVKMTTHLKKLKESYAQRQGVPMNSL[BzF]FLFEGQRIADNHTPKELGMEEEDVIEVYQEQTGG
Met-Gly-Cys-Ser-His$_6$-SUMO-1(C52A, L65BzF)	MGCSHHHHHHGSDQEAKPSTEDLGDKKEGEYIKLKVIGQDSSEIHFKVKMTTHLKKLKESYAQRQGVPMNSLRF[BzF]FEGQRIADNHTPKELGMEEEDVIEVYQEQTGG
Met-Gly-Cys-Ser-His$_6$-SUMO-1(C52A, F66BzF)	MGCSHHHHHHGSDQEAKPSTEDLGDKKEGEYIKLKVIGQDSSEIHFKVKMTTHLKKLKESYAQRQGVPMNSLRFL[BzF]EGQRIADNHTPKELGMEEEDVIEVYQEQTGG
Met-Gly-Cys-Ser-His$_6$-SUMO-1(C52A, E67BzF)	MGCSHHHHHHGSDQEAKPSTEDLGDKKEGEYIKLKVIGQDSSEIHFKVKMTTHLKKLKESYAQRQGVPMNSLRFLF[BzF]GQRIADNHTPKELGMEEEDVIEVYQEQTGG
Met-Gly-Cys-Ser-His$_6$-SUMO-1(C52A, G68BzF)	MGCSHHHHHHGSDQEAKPSTEDLGDKKEGEYIKLKVIGQDSSEIHFKVKMTTHLKKLKESYAQRQGVPMNSLRFLFE[BzF]QRIADNHTPKELGMEEEDVIEVYQEQTGG
Met-Gly-Cys-Ser-His$_6$-SUMO-1(C52A, Q69BzF)	MGCSHHHHHHGSDQEAKPSTEDLGDKKEGEYIKLKVIGQDSSEIHFKVKMTTHLKKLKESYAQRQGVPMNSLRFLFEG[BzF]RIADNHTPKELGMEEEDVIEVYQEQTGG
Met-Gly-Cys-Ser-His$_6$-SUMO-1(C52A, R70BzF)	MGCSHHHHHHGSDQEAKPSTEDLGDKKEGEYIKLKVIGQDSSEIHFKVKMTTHLKKLKESYAQRQGVPMNSLRFLFEGQ[BzF]IADNHTPKELGMEEEDVIEVYQEQTGG
Met-Gly-Cys-Ser-His$_6$-SUMO-1(C52A, E89BzF)	MGCSHHHHHHGSDQEAKPSTEDLGDKKEGEYIKLKVIGQDSSEIHFKVKMTTHLKKLKESYAQRQGVPMNSLRFLFEGQRIADNHTPKELGMEEEDVI[BzF]VYQEQTGG
Ubc9	MSGIALSRLAQERKAWRKDHPFGFVAVPTKNPDGTMNLMNWECAIPGKKGTPWEGGLFKLRMLFKDDYPSSPPKCKFEPPLFHPNVYPSGTVCLSILEEDKDWRPAITIKQILLGIQELLNEPNIQDPAQAEAYTIYCQNRVEYEKRVRAQAKKFAPS
MBP-PIASX-SIM-His$_6$	MKTEEGKLVIWINGDKGYNGLAEVGKKFEKDTGIKVTVEHPDKLEEKFPQVAATGDGPDIIFWAHDRFGGYAQSGLLAEITPDKAFQDKLYPFTWDAVRYNGKLIAYPIAVEALSLIYNKDLLPNPPKTWEEIPALDKELKAKGKSALMFNLQEPYFTWPLIAADGGYAFKYENGKYDIKDVGVDNAGAKAGLTFLVDLIKNKHMNADTDYSIA

	EAAFNKGETAMTINGPWAWSNIDTSKVNYGVTVLP TFKGQPSKPFVGVLSAGINAASPNKELAKEFLENYLL TDEGLEAVNKDKPLGAVALKSYEEELAKDPRIAAT MENAQKGEIMPNIPQMSAFWYAVRTAVINAASGRQ TVDEALKDAQTNSSSNNNNNNNNNNLGIEGRISEFL VPRGSKKVDVIDLTIESSSDEKVDHHHHHH
His_6-TEV-DPP9-S	MSYYHHHHHHDYDIPTTENLYFQGAMGSATTGTPT ADRGDAAATDDPAARFQVQKHSWDGLRSIIHGSRK YSGLIVNKAPHDFQFVQKTDESGPHSHRLYYLGMPY GSRENSLLYSEIPKKVRKEALLLLSWKQMLDHFQAT PHHGVYSREEELLRERKRLGVFGITSYDFHSESGLFL FQASNSLFHCRDGGKNGFMVSPMKPLEIKTQCSGPR MDPKICPADPAFFSFINNSDLWVANIETGEERRLTFC HQGLSNVLDDPKSAGVATFVIQEEFDRFTGYWWCP TASWEGSEGLKTLRILYEEVDESEVEVIHVPSPALEE RKTDSYRYPRTGSKNPKIALKLAEFQTDSQGKIVSTQ EKELVQPFSSLFPKVEYIARAGWTRDGKYAWAMFL DRPQQWLQLVLLPPALFIPSTENEEQRLASARAVPR NVQPYVVYEEVTNVWINVHDIFYPFPQSEGEDELCF LRANECKTGFCHLYKVTAVLKSQGYDWSEPFSPGE DEFKCPIKEEIALTSGEWEVLARHGSKIWVNEETKL VYFQGTKDTPLEHHLYVVSYEAAGEIVRLTTPGFSH SCSMSQNFDMFVSHYSSVSTPPCVHVYKLSGPDDDP LHKQPRFWASMMEAASCPPDYVPPEIFHFHTRSDVR LYGMIYKPHALQPGKKHPTVLFVYGGPQVQLVNNS FKGIKYLRLNTLASLGYAVVVIDGRGSCQRGLRFEG ALKNQMGQVEIEDQVEGLQFVAEKYGFIDLSRVAIH GWAYGGFLSLMGLIHKPQVFKVAIAGAPVTVWMA YDTGYTERYMDVPENNQHGYEAGSVALHVEKLPN EPNRLLILHGFLDENVHFFHTNFLVSQLIRAGKPYQL QIYPNERHSIRCPESGEHYEVTLLHFLQEYL
HA-DPP9	MASYPYDVPDYASLGSEFATTGTPTADRGDAAATD DPAARFQVQKHSWDGLRSIIHGSRKYSGLIVNKAPH DFQFVQKTDESGPHSHRLYYLGMPYGSRENSLLYSE IPKKVRKEALLLLSWKQMLDHFQATPHHGVYSREE ELLRERKRLGVFGITSYDFHSESGLFLFQASNSLFHC RDGGKNGFMVSPMKPLEIKTQCSGPRMDPKICPADP AFFSFINNSDLWVANIETGEERRLTFCHQGLSNVLDD PKSAGVATFVIQEEFDRFTGYWWCPTASWEGSEGL KTLRILYEEVDESEVEVIHVPSPALEERKTDSYRYPR TGSKNPKIALKLAEFQTDSQGKIVSTQEKELVQPFSS LFPKVEYIARAGWTRDGKYAWAMFLDRPQQWLQL VLLPPALFIPSTENEEQRLASARAVPRNVQPYVVYEE VTNVWINVHDIFYPFPQSEGEDELCFLRANECKTGF CHLYKVTAVLKSQGYDWSEPFSPGEDEFKCPIKEEIA LTSGEWEVLARHGSKIWVNEETKLVYFQGTKDTPL EHHLYVVSYEAAGEIVRLTTPGFSHSCSMSQNFDMF VSHYSSVSTPPCVHVYKLSGPDDDPLHKQPRFWAS MMEAASCPPDYVPPEIFHFHTRSDVRLYGMIYKPHA LQPGKKHPTVLFVYGGPQVQLVNNSFKGIKYLRLNT LASLGYAVVVIDGRGSCQRGLRFEGALKNQMGQVE

	IEDQVEGLQFVAEKYGFIDLSRVAIHGWSYGGFLSL<br>MGLIHKPQVFKVAIAGAPVTVWMAYDTGYTERYM<br>DVPENNQHGYEAGSVALHVEKLPNEPNRLLILHGFL<br>DENVHFFHTNFLVSQLIRAGKPYQLQIYPNERHSIRC<br>PESGEHYEVTLLHFLQEYL
HA-DPP9(V285A)	MASYPYDVPDYASLGSEFATTGTPTADRGDAAATD<br>DPAARFQVQKHSWDGLRSIIHGSRKYSGLIVNKAPH<br>DFQFVQKTDESGPHSHRLYYLGMPYGSRENSLLYSE<br>IPKKVRKEALLLLSWKQMLDHFQATPHHGVYSREE<br>ELLRERKRLGVFGITSYDFHSESGLFLFQASNSLFHC<br>RDGGKNGFMVSPMKPLEIKTQCSGPRMDPKICPADP<br>AFFSFINNSDLWVANIETGEERRLTFCHQGLSNVLDD<br>PKSAGVATFVIQEEFDRFTGYWWCPTASWEGSEGL<br>KTLRILYEEVDESEAEVIHVPSPALEERKTDSYRYPR<br>TGSKNPKIALKLAEFQTDSQGKIVSTQEKELVQPFSS<br>LFPKVEYIARAGWTRDGKYAWAMFLDRPQQWLQL<br>VLLPPALFIPSTENEEQRLASARAVPRNVQPYVVYEE<br>VTNVWINVHDIFYPFPQSEGEDELCFLRANECKTGF<br>CHLYKVTAVLKSQGYDWSEPFSPGEDEFKCPIKEEIA<br>LTSGEWEVLARHGSKIWVNEETKLVYFQGTKDTPL<br>EHHLYVVSYEAAGEIVRLTTPGFSHSCSMSQNFDMF<br>VSHYSSVSTPPCVHVYKLSGPDDDPLHKQPRFWAS<br>MMEAASCPPDYVPPEIFHFHTRSDVRLYGMIYKPHA<br>LQPGKKHPTVLFVYGGPQVQLVNNSFKGIKYLRLNT<br>LASLGYAVVVIDGRGSCQRGLRFEGALKNQMGQVE<br>IEDQVEGLQFVAEKYGFIDLSRVAIHGWSYGGFLSL<br>MGLIHKPQVFKVAIAGAPVTVWMAYDTGYTERYM<br>DVPENNQHGYEAGSVALHVEKLPNEPNRLLILHGFL<br>DENVHFFHTNFLVSQLIRAGKPYQLQIYPNERHSIRC<br>PESGEHYEVTLLHFLQEYL

Table S8: DPP9 plasmid information for expression in SF9 insect cells.

Name of construct	Plasmid name	Vector backbone
His_6-TEV-DPP9-short	Human DPP9-short	pFASTBacHT

Table S9: List of plasmids used for transfection.

Name of construct	Plasmid name	Vector backbone
HA-DPP9	pDPP9short	pcDNA3.1 (provided by (Pilla et al., 2012))
HA-DPP9(V285A)	pKBR119	pcDNA3.1

Supporting References

Aguilar-Martinez, E., Chen, X., Webber, A., Mould, A. P., Seifert, A., Hay, R. T., et al. (2015). Screen for multi-SUMO-binding proteins reveals a multi-SIM-binding mechanism for recruitment of the transcriptional regulator ZMYM2 to chromatin. *Prot. Natl. Acad. Sci. U. S. A.* 112, E4854-63. doi: 10.1073/pnas.1509716112

Cox, E., Hwang, W., Uzoma, I., Hu, J., Guzzo, C. M., Jeong, J., et al. (2017). Global Analysis of SUMO-Binding Proteins Identifies SUMOylation as a Key Regulator of the INO80 Chromatin Remodeling Complex. *Mol. Cell Proteomics* 16, 812–823. doi: 10.1074/mcp.M116.063719

Flotho, A., Werner, A., Winter, T., Frank, A. S., Ehret, H., and Melchior, F. (2012). Recombinant reconstitution of sumoylation reactions in vitro. *Methods Mol. Biol.* 832, 93–110. doi: 10.1007/978-1-61779-474-2_5

González-Prieto, R., Eifler-Olivi, K., Claessens, L. A., Willemstein, E., Xiao, Z., Talavera Ormeno, C. M. P., et al. (2021). Global non-covalent SUMO interaction networks reveal SUMO-dependent stabilization of the non-homologous end joining complex. *Cell Rep.* 34, 108691. doi: 10.1016/j.celrep.2021.108691

Pilla, E., Möller, U., Sauer, G., Mattiroli, F., Melchior, F., and Geiss-Friedlander, R. (2012). A novel SUMO1-specific interacting motif in dipeptidyl peptidase 9 (DPP9) that is important for enzymatic regulation. *J. Biol. Chem.* 287, 44320–44329. doi: 10.1074/jbc.M112.397224
